# Selected abstracts submitted to the Third International Symposium on Hereditary Breast and Ovarian Cancer: *BRCA:* Fifteen Years of Progress, Third International Symposium on Hereditary Breast and Ovarian Cancer; October 14–16, 2009; Centre Mont-Royal, Montreal, Quebec, Canada

**Published:** 2009-09

## Background

Germline mutation screening of *BRCA1* and *BRCA2* genes is performed in suspected familial breast cancer cases, but a causative mutation is found in only 30% of patients. The development of additional methods to identify good candidates for *BRCA1* and *BRCA2* analysis would therefore increase the efficacy of diagnostic mutation screening. With this in mind, we developed a study to determine molecular signatures of *BRCA1—*or *BRCA2—*mutated breast cancers.

## Materials and Methods

Array-cgh (comparative genomic hybridization) and transcriptomic analysis were performed on a series of 103 familial breast cancers. The series included 7 breast cancers with a *BRCA1* mutation and 5 breast cancers with a *BRCA2* mutation. The remaining 91 cases were obtained from 73 families selected on the basis of at least 3 affected first-degree relatives or at least 2 affected first-degree relatives with breast cancer at an average age of 45 years. Array-cgh analyses were performed on a 4407 BAC-array (CIT-V8) manufactured by IntegraGen. Transcriptomic analyses were performed using an Affymetrix Human Genome U133 Plus 2.0 chip.

## Results

Using supervised clustering analyses we identified two transcriptomic signatures: one for *BRCA1-*mutated breast cancers consisting of 600 probe sets and another for *BRCA2-*mutated breast cancers also consisting of 600 probes sets. We also defined cgh-array signatures, based on the presence of specific genomic rearrangements, one for *BRCA1-*mutated breast cancers and one for *BRCA2-*mutated breast cancers.

## Conclusions

This study identified molecular signatures of breast cancers with *BRCA1* or *BRCA2* germline mutations. Genes present in these signatures could be exploited to find new markers for such breast cancers. We also identified specific genomic rearrangements in these breast cancers, which could be screened for in a diagnostic setting using fluorescence *in situ* hybridization, thus improving patient selection for *BRCA1* and *BRCA2* molecular genetic analysis.

