# Supplementary material for: Selected abstracts submitted to the Third International Symposium on Hereditary Breast and Ovarian Cancer: BRCA: Fifteen Years of Progress, Third International Symposium on Hereditary Breast and Ovarian Cancer; October 14–16, 2009; Centre Mont-Royal, Montreal, Quebec, Canada
Source: Curr Oncol. 2009 Sep;16(5):91–110. (PMC2768503)
Supplement: Related texts

# Abstract: *P002* Identification and Characterization of Splice Variants in *BRCA* Genes Using Reverse Transcriptase Polymerase Chain Reaction and Multiplex Ligation-Dependent Probe Amplification

**Published:** 2009-09

**Authors:** R.D. Brandão, K. Van Roozendaal, D. Tserpelis, E. Goméz García, M.J. Blok

**Affiliations:** * Department of Clinical Genetics, University Hospital Maastricht and; † GROW – School for Oncology and Developmental Biology, Maastricht University, Maastricht, Limb, Netherlands

## Objectives

A large subset of the unclassified variants in the *BRCA1*/*2* genes are in intronic sequences or close to exon–intron boundaries and may affect splicing. Putative *BRCA1* and *BRCA2* splice variants, as predicted *in silico,* were studied experimentally using reverse transcriptase polymerase chain reaction (rt-pcr) and a new approach based on multiplex ligation-dependent probe amplification (mlpa) technology.

## Methods

Short-term lymphocyte cultures from controls and unclassified sequence variant carriers were established with pha and il-2. Analysis by rt-pcr was performed for the following *BRCA2* putative splice variants: c.425G>T, c.6935A>T, c.6842–3T>C, c.6943A>G, c.7976+3del2, c.8350C>T, c.8662C>T, c.8754+3G>C. The use of mlpa technology was subsequently evaluated for the detection of aberrant splice events on well-characterized splice variants, including *BRCA1* exon 13 and exon 22 deletions. Commercially available mlpa kits for *BRCA1* and *BRCA2* genes were used (probes in exons), in addition to homemade mlpa probe sets (probes crossing exon–exon boundaries).

## Results

*BRCA2* variants c.425G>T and c.7976+3del2 give rise to transcripts with deletions of exon 4 and exon 17 respectively, and c.8754+3G>C leads to 46 bp of intron retention. These variants were considered to be pathogenic. Additionally, alternative splicing events were observed in controls, such as c.6842_6937del (exon 12 skipping) and c.476_631del (exon 6 and 7 skipping). Because these in-frame transcripts are also expressed in healthy controls and have an unknown function, their clinical relevance remains unclear. This has consequences for the classification of c.6935A>T, which leads to increased expression of c.6842_6937del compared with controls. We found that, with mlpa, it is also possible to detect alternative splicing events.

## Conclusions

We characterized several new pathogenic *BRCA2* splice variants and demonstrated that, with mlpa technology, it is possible to set up a multiplex screen for semi-quantitative detection of alternative splice events that is also widely applicable for genes other than *BRCA1*/*2.*

# Abstract: *P003* Implementation of High Throughput Parallel Sequencing in a Diagnostic Setting: Multiplexed Amplicon Sequencing of the Breast Cancer Genes *BRCA1* and *2*

**Published:** 2009-09

**Authors:** K. De Leeneer, J. Hellemans, J. De Schrijver, W. Van Criekinge, A. De Paepe, P. Coucke, K. Claes

**Affiliations:** * Center for Medical Genetics, Ghent University Hospital and; † Laboratory for Bioinformatics and Computational Genomics, Ghent University, Ghent, Belgium

“Next generation” sequencing opens new perspectives for mutation analysis of large genes. We optimized a multi-sample barcode amplicon sequencing strategy for mutation detection in *BRCA1* and *2* on the gs-flx instrument (Roche/454 Life Sciences).

For the first two runs (POP1 and 2), 112 amplicons covering the complete coding sequence of *BRCA1* and *2* were pooled in equimolar amounts. Per run, 11 patients were sequenced in both directions for all amplicons. For a third run (POP3), all 112 amplicons were generated in 15 multiplex polymerase chain reaction (pcr) reactions. Hereby, all pcr reactions were optimized to obtain a final equimolar mix for all fragments (CV < 30%).

The sequence coverage was much more uniform in POP3 than in POP1 and POP2, indicating a successful optimization and showing that the bias introduced by the emulsion pcr is limited. In POP3, less than 40× coverage was obtained only for 6 amplicons. All sequence reads for the 22 patients of POP1 and POP2 were analyzed with our in-house variant identification pipeline (Deschrijver *et al.*, submitted) using the empirically determined filters (minimum coverage: 40×; maximum homopolymer length: 7; minimum variant frequency: 33%). These results were compared with data obtained by Sanger sequencing, producing 0 false negatives and 5 false positives. The filter settings applied for POP1 and POP2 are currently validated for POP3.

This approach is not yet cost-competitive with the high-resolution melting curve analysis (De Leeneer *et al.*, 2008, 2009) currently applied in our laboratory, but it outperforms traditional Sanger sequencing. Further optimization of sequence coverage uniformity, pooling of patients, and Titanium throughput will allow further cost reduction. For the optimization of next-generation amplicon sequencing, a strong collaboration is required between people involved in molecular diagnostics and bio-informaticians to obtain clinically reliable results.

# Abstract: *P005* Prevalence of *BRCA1*/*2* Mutations in Unselected and Selected French Canadian Breast Cancer Cases Diagnosed at the CRCHUM

**Published:** 2009-09

**Authors:** P. Ghadirian, A. Robidoux, J.F. Boileau, F.S. Côté, G. Martin, C. Maugard, E. Nassif, E. Patocskai, C. Potvin, R. Younan, W.D. Foulkes, S.A. Narod

**Affiliations:** * Centre hospitalier de l’Université de Montréal (crchum) and; † McGill University, Montreal, Quebec, and; ‡ University of Toronto, Toronto, Ontario, Canada

## Objectives

The main purpose of this study was to estimate the prevalence of *BRCA* gene mutations and the yield of genetic testing among unselected breast cancer patients less than 50 years of age compared with selected patients between 51 and 65 years of age (with a family history of 2 or more breast or ovarian cancers).

## Methods

In an open-cohort clinic at the crhum in which French-Canadian breast cancer patients from 2005–2009 were recruited, we studied 932 subjects (687 < 50 years of age and 245 > 50 years of age). All subjects were tested for *BRCA* gene mutations.

## Results

Of the unselected women with breast cancer (<50 years of age), 5.0% were *BRCA* mutation carriers (1.2% *BRCA1,* 3.8% *BRCA2*). In those with a family history (selected subjects or >50 years of age), 4.9% displayed *BRCA* mutations (0.8% *BRCA1,* 4.1% *BRCA2*). Among unselected patients, the refusal rates for the genetic tests were 10.2% and 14.9% among selected breast cancer patients. Of the study subjects, 99% agreed to be told the results of their genetic tests; the remaining 1% refused to be told their results.

## Conclusions

The results of this open-cohort study of the genetic epidemiology of breast cancer showed that the prevalence of *BRCA* mutations among French-Canadian breast cancer patients is around 5.0% (5.0% in unselected or <50-year-old patients, 4.9% in selected or older subjects). This type of study is feasible for genetic screening and the refusal rate for participation in a genetic study of breast cancer is very low.

# Abstract: *P006* The Establishment of a Cohort of Girls in Ontario Breast Cancer Families

**Published:** 2009-09

**Authors:** D. Hanna, G. Glendon, E. Maloney, J. Knight, L. Lilge, D. Koller, I. Andrulis

**Affiliations:** * Cancer Care Ontario; † University Health Network; ‡ Mount Sinai Hospital and; § The Hospital for Sick Children Toronto, Ontario, Canada

## Background

Emerging evidence indicates that childhood and adolescence may be periods sensitive to factors associated with breast cancer initiation or protective against breast cancer development. To investigate factors related to breast cancer risk, we are planning a study of girls. The legacy (Lessons in Epidemiology and Genetics of Adult Cancer from Youth) study plans to examine several intermediate phenotypes associated with breast cancer, including pubertal breast development, breast tissue characteristics, age at menarche, sex and growth hormone levels, and their relationship with potentially modifiable early-life exposures, as well as genetic determinants.

## Objectives

To determine the interest of parents and daughters for participating in legacy and to aid in its design, we are conducting a pilot study to evaluate the feasibility of recruitment, biospecimen, and data collection in 100 girls; to determine whether, as compared with Tanner stage, optical spectroscopy can be used as an efficient measure of breast development in the girls; and to define parental and offspring levels of acceptance or dissatisfaction across data-collection measures.

## Methods

Parents and their 6- to 17-year-old daughters from two high-risk cancer genetics clinics in the Greater Toronto Area and those already enrolled in the Ontario Familial Breast Cancer Registry, a U.S. National Cancer Institute–supported population-based registry, will be invited to participate. The pilot involves the completion of self- and interview-administered epidemiologic questionnaires for daughter and parent; clinic-administered measures of height and weight; blood, buccal, and urine sampling; measures of pubertal breast development using self–Tanner staging and experimental optical spectroscopy modalities; and qualitative exit interviews for thorough exploration of participant likes and dislikes.

## Results

We will present recruitment outcomes from our first group of girls.

## Conclusions

Completion of the pilot study will allow us to demonstrate feasibility, to estimate response rates, and to assess the interest of families in participating in legacy.

# Abstract: *P007 BRCA1* and *BRCA2* Mutations in Belgian Families with Breast and/or Ovarian Cancers

**Published:** 2009-09

**Authors:** P. Hilbert, A. Destrée, F. Cavallin, C. Verellen–Dumoulin

**Affiliations:** Human Genetics Center, Institut de Pathologie et Génétique, Charleroi, Belgium

Germline mutations in *BRCA1* and *BRCA2* genes cause predisposition to breast and ovarian cancers. Here, we describe the experience of our Human Genetics Center on a total of approximately 700 patients living in the southern part of Belgium. These patients, apparently from independent families, were referred by oncologists or by geneticists. All of them had a personal and/or familial history suggestive of genetic predisposition to breast and/or ovarian cancer.

The entire coding regions of the *BRCA1* and *2* genes were screened by standard scanning methods (denaturing gradient gel electrophoresis, denaturing high-pressure liquid chromatography, or high resolution melt), followed by sequencing of abnormal profiles. Large rearrangements of the two genes were investigated by mlpa (multiple ligation-dependent probe amplification from MRC-Holland).

A total of 186 variants (excluding known polymorphisms) were identified (106 in *BRCA1,* 80 in *BRCA2*). This represents a detection rate of 26.5%.

In *BRCA1*, 26 (24.5%) were variants of uncertain significance, and 80 (75.5%) were clear mutations, of which 13 were large rearrangements of the gene. Four recurrent mutations (deletion from exons 8 to 13, c.2722G>T, c.4391_4393delinsTT, and c.5137del) account for 27.5% (respectively 7.5%, 7.5%, 6.25%, and 6.25%) of the mutations. In *BRCA2*, 37 (46%) were variants of uncertain significance, and 43 (54%) were clear mutations, of which only 1 was a large rearrangement of the gene. Two recurrent mutations (c.9117G>A and c.4936_4939del) account for 30% (respectively 16.3% and 14%) of the mutations.

A causal mutation has hence been identified in a total of 17.5% of the patients (11.4% in *BRCA1,* 6.1% in *BRCA2*). These results underline the role of large rearrangements of *BRCA1* (16.25% of the mutations identified in this gene) in Belgium and point to the need for collaborative efforts to classify precisely variants of uncertain significance, because they represent 33.8% of all variants detected.

# Abstract: *P008* Breast and Ovarian Cancer Risk Among Heterozygotes for Xeroderma Pigmentosum Nucleotide Excision Repair Gene Mutations

**Published:** 2009-09

**Authors:** R. Moslehi

**Affiliations:** State University of New York at Albany, New York, and National Institutes of Health, Bethesda, Maryland, U.S.A

## Background

Xeroderma pigmentosum (xp) is a rare autosomal recessive disorder with an estimated frequency of 10–6 in North America. The condition is characterized by severe photosensitivity, abnormal pigmentation, and an increase, by a factor of 1000 or more, in the risk of cancers in sun-exposed tissues (skin and eye) in affected individuals. The disorder is caused by mutations in a number of genes, all (*XPA–XPG*) but one (*XP-*variant) involved in the nucleotide excision repair (ner) pathway. The heterozygote (clinically normal-appearing carrier) frequency of *XP* gene mutations is estimated at about 1 in 300. Cancer risk of heterozygotes is not known. We report on the frequency of hereditary breast cancer among xp families.

## Methods

The study population consists of 22 xp families with detailed multi-generation pedigrees and epidemiology information ascertained under a protocol to study the natural history of the disorder. Hereditary breast cancer was defined as the occurrence of at least 2 cases of premenopausal breast cancer (diagnosed <50 years of age) or 1 case of premenopausal breast cancer and 1 case of ovarian cancer in one side of the family.

## Results

The family histories of 5 of the 22 xp patients were compatible with hereditary breast cancer. In xp family R1, with causative mutations in *XPA,* there were 4 cases (including the heterozygote mother of the proband) of premenopausal breast cancer, 1 case of ovarian cancer, and 1 case of prostate cancer in the maternal side. Comprehensive sequencing failed to identify a mutation in the *BRCA1* and *BRCA2* genes in this family. Overall, a family history compatible with hereditary breast cancer was found in approximately 23% of xp families reported here.

## Conclusions

Heterozygote carriers of *XP* gene mutations may be at increased risk for breast and ovarian cancer. A proportion of breast and ovarian cancer cases in the population may be attributed to mutations and polymorphisms in the ner genes.

# Abstract: *P009* Improvement in the Detection Rate of the *BRCA1*/*BRCA2* French-Canadian Mutation Panel at the Service de Médecine Génique, CHUM

**Published:** 2009-09

**Authors:** S. Nolet, K. Latour, S. Côté, N. Dumas, M.C. Binet, M. Breguet, L. Gaboury, P. Hamet, N. Larouche, G. Proulx, D. Provencher, J. Richard, C. Wilmart, C. Maugard

**Affiliations:** Centre hospitalier de l’Université de Montréal (chum), Montreal, Quebec, Canada

## Introduction

The genetics of the French-Canadian (fc) population of Quebec is shaped by well-documented founder effects that have allowed for the identification of common mutations in specific genes. In *BRCA1* and *BRCA2* hereditary breast and ovarian cancer, several mutations are part of a fc panel which is used as a first line of testing.

## Objectives

To evaluate the detection rate of the fc panel currently used in the chum diagnostic laboratory. To determine the change in detection rate when mutations, reported in at least two distinct fc families, are added to the panel.

## Methods

A retrospective review of results obtained for fc families tested through the Hereditary Cancer Clinic using the laboratory and clinical records. Included in our analysis were index cases for whom the affected branch of the family was of fc descent, for whom a whole blood sample could be obtained, and who had both panel testing and sequencing if panel testing was negative (*n* = 301).

## Results

From January 2005 to December 2008, 408 cases of fc descent were screened for 9 mutations (R1443X, 2953del3+C, 3768insA, W321X for *BRCA1*; 8765delAG, E1953X, 3398del5, 2816insA, 6503delTT for *BRCA2*). Of the 301 index cases corresponding to our inclusion criteria, 69 were identified as mutation carriers. Of these 69 carriers, 57 were found to carry a mutation from the fc panel (82.6%). The remaining 12 had a mutation identified by sequencing (17.4%), and 5 of these mutations (2080insA, 2244insA, 2800delAA for *BRCA1*; 3773delTT, r2336H for *BRCA2*) were reported in at least 2 distinct families. By adding these mutations to our in-house panel, our detection rate will reach 97.1% (67/69).

## Conclusions

This retrospective review of our laboratory data indicates that the detection rate of our in-house fc panel of 9 mutations is 82.6%. By adding 5 mutations, this detection rate will reach 97.1%. These preliminary data indicate that testing using the fc panel in appropriate families continues to be a cost-effective initial screening. The data are insufficient to preclude further testing by sequencing in panel-negative families. A periodic review of mutations identified by sequencing in fc families is important to improve and validate the efficiency of the fc panel.

# Abstract: *P010* The Characterization of Genomic Instability in Lymphoblastoid Cell Lines Derived from Heterozygous *PALB2* Mutation Carriers

**Published:** 2009-09

**Authors:** D.J. Novak, L. Amrein, J. Reddy, L. Wark, R. Aloyz, S. Mai, M.D. Tischkowitz

**Affiliations:** * Program in Cancer Genetics; † Medicine and Human Genetics and; ‡ Segal Cancer Center, McGill University, Montreal, Quebec and; § Institute of Cell Biology, University of Manitoba, Winnipeg, Manitoba, Canada

## Objectives

*PALB2,* upon interaction with its nuclear partner, the breast cancer gene, *BRCA2,* is known to promote localization and stability. *PALB2* is estimated to confer a moderately increased risk for breast cancer, likely because of a disruption of genomic regulation. To date, the underlying pathogenesis of *PALB2* mutations has yet to be illuminated by common molecular mechanisms. It has previously been suggested that Fanconi anemia proteins, a group of which *PALB2* is a member, may play a critical role in the telomere maintenance pathway, because of their core function in genetic recombination. In the current study, we attempt to establish both a molecular and cellular link between *PALB2* heterozygous mutations and genomic instability, an important precursor to carcinogenesis.

## Methods

Six lymphoblastoid cell lines (lcls) were analyzed in the current study, two expressing wild-type *PALB2,* and four carriers of distinct heterozygous *PALB2* truncating mutations: 2521delA, 3323delA, Q775X, and 229delT. Genomic integrity was assessed by telomeric quantitative fluorescence *in situ* hybridization (q-fish). Further investigations of genomic abnormalities were conducted through the use of centromeric fish and spectral karyotyping. The response to cellular cytotoxicity was examined in the presence of mitomycin C, bleomycin, and cisplatin. Cellular survival was measured via photo spectrophotometry through the metabolism of the tetrazolium salt WST-1.

## Results

No statistical difference was observed in telomere number or median telomeric intensity between the two *PALB2* control lines (C1 and C2, *p* = 0.287). Similarly no significant differences were observed in telomere number or intensity between C1/C2 and the 3323delA carrier (*p* = 0.386 vs. C1 and *p* = 0.786 vs. C2). Probe intensity was markedly reduced in the 2521delA carrier as compared with both C1 and C2. The Q775X carrier was observed with a reduced telomere count and differed significantly from either control with respect to mean probe intensity (*p* = 2.22^−12^ vs. C1 and *p* = 8.52^−15^ vs. C2). Similarly, the 229delT carrier was observed with an increased telomere count and significantly differed in mean probe intensity (*p* = 6.05^−17^ vs. C1 and *p* = 1.77^−12^ vs. C2). No significant differences were observed in centromere number throughout all six lcls. No duplications, translocations, deletions, or rearrangements were observed to be consistent with the pathogenicity of the *PALB2* variants. No lcls showed any significantly altered response to any of the cytotoxic agents.

## Conclusions

The current study may suggest a possible association between *PALB2* heterozygous truncating mutations and impaired genomic regulation. However, a new model system for analysis, such as a *PALB2* tumour cell line, is required to further elucidate this potential association.

# Abstract: *P011* Frequency of French-Canadian (FC) *BRCA1*/*2* Common Founder Mutations in FC Hereditary Breast/Breast and Ovarian Families Evaluated in Ottawa, Ontario

**Published:** 2009-09

**Authors:** E. Smith, E. Tomiak, K. Teed, E. McCready, N. Carson

**Affiliations:** Children’s Hospital of Eastern Ontario, Ottawa, Ontario, Canada

## Objectives

Previous research has demonstrated that most *BRCA1*/*2* mutations (84%) detected in French-Canadian (fc) hereditary breast or breast/ovarian cancer families living in Quebec are accounted for by 1 of 8 recognized common founder mutations (Tonin *et al. Bull Cancer* 2006:93:841–6). The objective of our study was to describe the pattern of *BRCA1* and *2* mutations identified by the Molecular Genetics Laboratory of the Eastern Ontario Regional Genetics department located in Ottawa, Ontario.

## Methods

Between April 1998 and May 2009, a retrospective chart review was conducted to obtain ethnicity data in women identified to carry a pathogenic *BRCA1*/*2* mutation. The charts of 112 index cases were available for review. In 22 individuals, information regarding ancestry was missing or ancestry was unknown, leaving 90 cases available for analysis. All cases represented the first family member to be tested in our laboratory and found to carry a pathogenic mutation.

## Results

The fc founder mutations were identified in 13/15 (86.7%) of families whose ethnicity was identified as fc and in additional 8/12 families where ancestry was considered to be probably or possibly fc. Among the 22 families with missing/unknown ancestry, an additional 2 fc founder mutations were identified, for a total of 23 index cases found to carry a common fc founder mutation.

## Conclusions

Our findings in a regional laboratory which serves both Franco-Ontarians and Québécois francophones parallel the demonstration of a limited spectrum of pathogenic *BRCA1*/*2* mutations in Quebec patients previously reported by Tonin *et al.* The high percentage of cases accounted for by a limited panel of 8 common founder mutations suggests that consideration be given in Ontario to the evaluation of initial screening with a mutation panel in patients of French-Canadian ancestry.

# Abstract: *P012* Establishing the Evidence Base to Translate *PALB2* Mutation Testing into Breast Cancer Clinical Genetics Services

**Published:** 2009-09

**Authors:** M. Southey, M. Tischkowitz, Z.L. Teo, F. Odefrey, N. Sabbaghian, G. Byrnes, I. Winship, M. McCredie, G. Giles, D. Goldgar, W. Foulkes, J. Hopper

**Affiliations:** * The University of Melbourne, Victoria, Australia; † McGill University and; ‡ Sir Mortimer B. Davis Jewish General Hospital, Montreal, Quebec, Canada; § The International Agency for Research on Cancer, Lyon, France; || The University of Otago, Dunedin, New Zealand; # The Cancer Council Victoria, Victoria, Australia; ** University of Utah School of Medicine, Salt Lake City, Utah, U.S.A.; †† Breast Cancer Family Registry, National Cancer Institute, National Institutes of Health, Bethesda, Maryland, U.S.A. and; ‡‡ Peter MacCallum Cancer Centre, Victoria, Australia

Mutations in *PALB2* have been reported to be associated with a doubled to tripled increased risk of breast cancer. Women with mutations in *PALB2* who also have a strong family history of breast cancer are predicted to be at a high absolute risk for the disease comparable to that in women who carry *BRCA2* mutations. A current paucity of information about *PALB2* mutation carrier risks prevents testing for mutations in this gene being incorporated into evidenced-based clinical genetics services.

## Objectives

To conduct a large case–control family study using population-based and clinic-based resources, to characterize *PALB2* mutations in terms of breast cancer risk, and to devise criteria to identify women most likely to carry *PALB2* mutations.

## Methods

High-resolution melt curve analysis and Sanger sequencing.

## Results

To date, we have screened 756 early-onset population-based cases for *PALB2* mutations and identified 2 carriers, both in women with extremely strong family histories of breast cancer. We have also screened 400 women from multiple-case breast cancer families and identified 4 families that carry *PALB2* mutations. Testing for these mutations in relatives has identified an additional 20 *PALB2* mutation carriers. Breast cancers arising in affected female carriers are commonly grade 3 (7/12 reviewed to date) and estrogen receptor–positive (7/9 reviewed to date); 9 are infiltrating ductal carcinomas, and 3 are lobular or pleomorphic lobular carcinomas.

## Conclusions

We and others have produced preliminary and circumstantial evidence that women who carry a mutation in *PALB2* are at an elevated risk of breast cancer, and that depending on family history, these risks could be of clinical relevance comparable to that for women with *BRCA2* mutations. *PALB2* mutations are rare, but for the women who carry these mutations and their families, it is potentially important that mutations be identified so that the women can be offered appropriate prevention, screening, and clinical management.

# Abstract: *P013* Recurrent *BRCA1* Mutations Identified in Manitobans of Eastern European Descent^a^

**Published:** 2009-09

**Authors:** E. Spriggs, T. Dyck, R. Jobse, B. Chordirker, A. Chudley, S. Marles, K. Serfas, Julie Richer

**Affiliations:** * Clinical Biochem and Genetics, Diagnostic Services of Manitoba; † Department of Biochemistry and Medical Genetics and; ‡ Department of Pediatrics and Child Health, University of Manitoba and; § Genetics and Metabolism Program, Winnipeg Regional Health Authority, Winnipeg, Manitoba and; || Medical Genetics, Children’s Hospital of Eastern Ontario, Ottawa, Ontario, Canada

Approximately 1 in 5 people living in the Canadian province of Manitoba are of Eastern European descent, with Ukraine being the most frequently named country of origin. Mutations in *BRCA1* and *BRCA2* account for most hereditary breast and ovarian cancers. Ten years ago, testing for *BRCA1* and *BRCA2* mutations in Manitoba was limited to the three common mutations found in the Ashkenazi Jewish population, and shortly thereafter included screening by protein truncation testing. Through results obtained in-house and from published data, it became recognized that one of the three common mutations, *BRCA1* c.5266dupC (5382insC), was also common in individuals of European descent, particularly those from Eastern Europe. Twelve unrelated Manitoban families were identified with the c.5266dupC mutation over the first 8 years of *BRCA1* and *BRCA2* genetic testing in Manitoba, and of these, only 4 (30%) are from families of Ashkenazi Jewish descent. The remaining cases were found in families reported to be of Ukrainian (*n* = 5), Polish (*n* = 1), Hungarian (*n* = 1), and German/unknown (*n* = 1) descent, with no known Jewish background. Given this observation and the relatively high proportion of the Manitoba population being of Eastern European descent, an ethnic-specific panel targeted to individuals of Eastern European descent was created. It included three *BRCA1* mutations (c.5266dupC, p.Cys61Gly, c.4034delA) that were described in literature as “recurrent” for this population. Over the past 3 years, 55 Manitobans have been tested, and two new families with mutations in *BRCA1* were found, one with c.5266dupC and one with p.Cys61Gly. The c.5266dupC case, representing our 13th family, was identified in a woman of Ukrainian/German Mennonite descent who had bilateral breast cancer. The p.Cys61Gly was found in a Ukrainian family with a history of breast and ovarian cancer. A second unrelated family of Ukrainian descent was identified with the p.Cys61Gly mutation through denaturing high-pressure liquid chromatography mutation scanning of the *BRCA1* and *BRCA2* genes. As part of our full screen available to affected patients who meet our testing criteria for *BRCA1* and *BRCA2,* a novel and yet recurrent *BRCA1* genomic deletion involving exons 13 through 19 was identified in 3 unrelated Manitoban families, with Ukrainian descent being the only common theme. Data accumulated thus far suggest that the founder *BRCA1* mutations for Manitobans of Eastern European descent are similar to those described by others, c.5266dupC and p.Cys61Gly, and that this unique in-frame deletion involves exons 13–19.

# Abstract: *P014* Identification of a *BRCA2* Founder Mutation in the Manitoba Mennonite Population^b^

**Published:** 2009-09

**Authors:** E. Spriggs, K. Serfas, S. Burgess, B. Chodirker, A. Chudley, S. Marles, T. Dyck

**Affiliations:** * Clinical Biochem and Genetics, Diagnostic Services of Manitoba; † Department of Biochemistry and Medical Genetics and; ‡ Department of Pediatrics and Child Health, University of Manitoba and; § Genetics Program, Winnipeg Regional Health Authority, Winnipeg, Manitoba, Canada

The Mennonites are a distinct ethnic and religious group characterized by their Anabaptist beliefs and pacifism. Over the last few centuries, persecution forced this group to migrate throughout Northern and Eastern Europe, and then to Southern Manitoba (Canada), the American Midwest, Mexico, and Central and South America. There are about 66,000 Mennonites residing in the Canadian province of Manitoba, accounting for approximately 6% of its total population. Founder mutations have been identified in the Mennonite population for a number of genetic diseases, including hypophosphatasia, X-linked congenital stationary night blindness, and familial hypertrophic cardiomyopathy. We have identified a mutation, c.5238dupT (p.Asn1747fs), in the *BRCA2* gene in 4 unrelated Mennonite families. The index patients were referred for genetic testing of *BRCA1* and *BRCA2* genes because of personal and family histories of breast and ovarian cancer. One family also had a history of pancreatic cancer. Of the 4 families, 3 were long-time residents of Manitoba; 1 family had recently emigrated from Bolivia. Further investigations into the pedigrees did not reveal any common relatives between the families. These mutations were independently identified by dna sequencing following the observation of a truncated band using the protein truncation test targeted to exon 11 of *BRCA2*. The c.5238dupT mutation has been reported only a limited number of times in the Breast Cancer Information Core database and in the literature, and it has been restricted to patients of Dutch, German, Mennonite, and Western and Eastern European descent. The finding of 4 large pedigrees of Mennonite descent indicates the presence of a founder mutation in the Manitoba Mennonite population. The population frequency of this *BRCA2* c.5238dupT mutation among Manitoba Mennonites is not known, but it may be of value to offer targeted mutation analysis to individuals of Mennonite descent who have a personal and/or family history of breast and ovarian cancer. However, it is important to note that other mutations, such as *BRCA2* c.6952C>T (p.Arg2318X), have been identified in Manitoba Mennonites with strong family histories of cancer. Such families that meet testing criteria and do not carry the c.5238dupT mutation should be offered full screening for *BRCA1* and *BRCA2*.

# Abstract: *P015* The Breast Cancer Genes International Agency for Research on Cancer Database: A Tool to Improve Evaluation of *BRCA1* and *BRCA2* Unclassified Sequence Variants

**Published:** 2009-09

**Authors:** M. Vallee, F. Lesueur, A. Spurdle, F. Hogervorst, D.E. Goldgar, S.V. Tavtigian

**Affiliations:** * International Agency for Research on Cancer, Lyon, France; † Queensland Institute of Medical Research, Herston, Queensland, Australia; ‡ The Netherlands Cancer Institute, Amsterdam, Netherlands and; § University of Utah School of Medicine, Salt Lake City, Utah, U.S.A

*BRCA1* and *BRCA2* are the two major high-risk breast cancer susceptibility genes, and the scale of clinical mutation screening of these genes probably exceeds that of any other set of cancer susceptibility genes. One complication resulting from genetic testing is that between 5% and 15% of patients screened are found to carry an unclassified sequence variant (uv). Observations of uvs create problems for the clinical lab staff who initially detect the uvs through to the patients who receive the ambiguous information.

The Breast Cancer Information Core has developed a Bayesian integrated evaluation of uvs based on co-segregation, personal and family history, tumour histopathology, and co-occurrence of variants in trans. Integrated evaluation includes a prior probability based on these *in silico* analyses of the substitutions, both from the perspective of potential effects on mrna splicing and potential effects on protein function. To facilitate the evaluation of *BRCA1* and *BRCA2* uvs, we created the BRCA International Agency for Research on Cancer (iarc) database. This database contains the set of all possible single nucleotide substitutions (which constitute a sizeable preponderance of all uvs) to the open reading frames of *BRCA1* and *BRCA2. In silico* analysis programs can be run on this finite set. The *BRCA* iarc database summarizes predicted potential to interfere with mrna splicing (MaxEnt and NNSplice) and predicted missense substitution severity (position and Align-GVGD, using curated protein multiple sequence alignments) on the entire set. These scores are integrated to give a prior probability of pathogenicity for each substitution. Thus the database, and the Web site through which we present it (brca.iarc.fr/), provides a starting point for the integrated evaluation of most *BRCA1* and *BRCA2* uvs.

# Abstract: *P016* Pathogenicity of the *BRCA1* Missense Variant D1739V Supported by Multiple Lines of Evidence

**Published:** 2009-09

**Authors:** R.B. van der Luijt, A.N.A. Monteiro, A.Y. Bronkhorst, T.G.W. Letteboer, C.J.M. van der Sijs–Bos, J.G. Post, R. Karchin, S.V. Tavtigian, D.E. Goldgar, P.M. Nederlof, H.K.P. van Amstel

**Affiliations:** * Department of Medical Genetics, University Medical Center, Utrecht, Netherlands; † H. Lee Moffitt Cancer Center and Research Institute, Tampa, Florida and; ‡ Department of Biomedical Engineering, Johns Hopkins University, Baltimore, Maryland, U.S.A.; § International Agency for Research on Cancer, World Health Organization, Lyon, France; || Department of Dermatology, University of Utah School of Medicine, Salt Lake City, Utah, U.S.A. and; # Department of Pathology, Netherlands Cancer Institute, Amsterdam, Netherlands

## Objectives

The clinical relevance of most non-truncating mutations in the *BRCA1* and *BRCA2* genes, including amino acid substitutions, is unknown. The assessment of these unclassified variants (uvs), which is of crucial importance for appropriate risk management, requires a multimodal approach.

## Methods

By integrating classical genetic, tumour histopathologic, functional, evolutionary conservation, and computational approaches, we investigated the pathogenic nature of a missense mutation, D1739V (c.5216A>T, p.Asp1739Val), of the *BRCA1* gene identified in 2 of our breast/ovarian cancer families.

## Results

The D1739V variant showed complete co-segregation in both families, loss of the wild-type *BRCA1* allele with retention of the variant allele in tumour-derived dna, a *BRCA1-*like genomic profile in a breast tumour from an uv carrier, and loss-of-function in transcriptional activity assays. D1739 is located within the brct domain of *BRCA1*. A protein multiple sequence alignment containing 13 *BRCA1* orthologs from different species showed complete evolutionary conservation of D1739. Computational analysis using the Align-GVGD method classifies D1739V as highly likely to interfere with normal protein function, based on the evolutionary conservation of D1739 and physicochemical characteristics of the amino acid pair D and V. The functional impact of the D1739V variant was also analyzed by three supervised learning programs, all predicting a deleterious effect. Protein modeling of the brct domain of *BRCA1* suggested that D1739V disrupts the conformational stability of this domain.

## Conclusions

All types of data generated in this study support a pathogenic nature of the variant D1739V, and we therefore propose to classify this amino acid substitution as a deleterious *BRCA1* mutation.

# Abstract: *P017* Hereditary Breast/Ovarian Cancer and *BRCA* Mutations: A South African Perspective

**Published:** 2009-09

**Authors:** N.C. van der Merwe, E. Jansen van Rensburg

**Affiliations:** * University of the Free State and; † National Health Laboratory Services, Bloemfontein, Free State and; ‡ University of Pretoria, Pretoria, Gauteng, South Africa

## Objectives

Breast cancer is the most common cancer to affect South African women, with a lifetime risk of 1 in 13 for the Afrikaner. Discovery of the *BRCA* genes introduced a new era for genetic testing, for not only could affected patients be informed regarding their risk for other cancer types, but other at-risk relatives could also be identified. The discovery of these genes urged many countries, including South Africa, to launch investigations into the prevalence of mutations in these genes.

## Methods

A total of 157 Afrikaner breast and/or ovarian cancer families (≥3 cases) were screened using conventional polymerase chain reaction analyses. Families carrying identical mutations were genotyped to determine whether the mutations resulted from independent events or a common ancestor. A genetic counselling protocol, based on classical clinical counselling strategies, was developed for patients requesting testing.

## Results

*BRCA1* mutations accounted for 19% (30/157) and *BRCA2* mutations for 47% (73/157) of mutation-positive families. Three recurrent mutations were identified, two in *BRCA1*, 1493delC and E881X, and one in *BRCA2*, 8162delG. Genotype and genealogical analyses indicated common ancestors, with a specific European founding couple identified for each. Collectively, the 3 Afrikaner founder mutations accounted for 94% (97/103) of *BRCA* mutation-positive families. A diagnostic testing protocol has since been implemented. Because of incomplete penetrance, preliminary investigations into potential single-nucleotide polymorphisms revealed that homozygosity for the variant allele of *WAF1* IVS2+16C→G proved to be associated with an increased breast cancer risk among *BRCA2* mutation carriers affected with breast cancer specifically.

## Conclusions

The 3 founder mutations are unique and limited to the South African Afrikaner population. The uniqueness could possibly be attributable to both mutations being *de novo* occurrences in one of the founders; or the particular mutations might be extremely rare in the European populations from which the forefathers originally came; or lastly, both mutations were initially present in the European populations, but have become extinct.

# Abstract: *P018* Frequency of the c.1100delC *CHEK2* Mutation in a Cohort of Belgian Familial Breast Cancer Patients: The Leuven Experience

**Published:** 2009-09

**Authors:** G. Michils, M. Chmara, G. Matthijs, E. Legius

**Affiliations:** Center for Human Genetics, University of Leuven, Leuven, Belgium

Nowadays genetic testing for hereditary breast cancer is offered worldwide. In about 20% of familial cases, the disease-causing mutations can be identified; the rest remain unexplained. Many of these mutations occur in the high-penetrance breast cancer genes *BRCA1* and *BRCA2*. A limited percentage are attributable to pathogenic mutations in moderate-penetrance genes such as *CHEK2*.

## Objectives

In Leuven, the familial cases are routinely tested for mutations in the *BRCA1*/*2* genes. To study the involvement and the implications of the specific c.1100delC *CHEK2* variant in breast cancer, a large cohort of affected patients has been tested for the presence of this mutation. The collected data have been analyzed and discussed for possible further clinical service.

## Methods

The presence of the c.1100delC mutation was assessed by multiplex ligation-dependent probe amplification (P-045 MRC-Holland). The positive samples have been subsequently sequenced for confirmation.

## Results

The frequency of the c.1100delC mutation in Belgian patients is 3.4%—very close to the frequency found in the Netherlands. Compared with *BRCA1*/*2* mutations, this *CHEK2* mutation was more frequent in bilateral breast cancer patients and in patients with an age of onset over 46 years. Our data are also consistent with the observation that the *CHEK2* gene is a multi-organ gene.

## Conclusions

Although the *CHEK2* gene is a moderate-risk breast cancer gene, these data can be taken into account because of the implications for clinical practice. Given that the relative risk of breast cancer for *CHEK2* by age 60 is 6%–10% (vs. 30%–60% for *BRCA1*/*2*), clinical follow-up for patients carrying the c.1100delC mutation has been adapted to become more stringent. The frequency of this mutation in Belgium warrants its implementation in the diagnostic service.

# Abstract: *P020* Clinical, Pathologic, and Genetic Characteristics of Chinese Patients with *BRCA-*Related Breast Cancer

**Published:** 2009-09

**Authors:** A. Kwong, E.K.O. Ng, C.L.P. Wong, E.S.K. Ma

**Affiliations:** * Division of Breast Surgery, The University of Hong Kong Li Ka Shing Faculty of Medicine and; † Cancer Genetics Centre and; ‡ Division of Molecular Pathology, Hong Kong Sanatorium and Hospital, Hong Kong SAR, PR China and; § Department of Surgery, Stanford University, Stanford, California, U.S.A

## Background

Breast cancers attributable to underlying germline *BRCA1* and *BRCA2* mutations are associated with particular pathologic features that may differ from those of sporadic breast cancers. We report clinical and pathologic characteristics of breast cancer in Chinese women with and without mutations and of carriers of *BRCA1* mutations as compared with *BRCA2* mutations.

## Methods

Based on age and family history, 226 high-risk women were recruited from March 2007 to November 2008. Medical information was prospectively collected from the patients and medical records. *BRCA1* and *BRCA2* mutations were detected using full gene sequencing and multiplex ligation-dependent probe amplification.

## Results

Of the 226 female probands tested, 28 women (12.4%) were *BRCA* mutation carriers, and among these carriers, 11 (39.3%) had *BRCA1* and 17 (60.7%) had *BRCA2* mutations. The *BRCA* mutation carriers were more likely to have a familial history of breast and ovarian cancer, high-grade cancers, and triple negative (tn) cancers. Prevalence of tn was 48.3% in *BRCA* carriers and 25.6% in non-carriers; it was 67.7% in *BRCA1* and 35.3% in *BRCA2* carriers. Estrogen receptor–negative cancer was significantly associated with *BRCA1* mutations, especially in those under 40 years of age (odds ratio: 5.14; confidence interval: 1.03 to 25.60). In this cohort, one recurrent *BRCA2* mutation was found in 4 probands, accounting for 23.5% of *BRCA2* mutations and 14.3% of all *BRCA* mutations. The haplotype analysis confirmed the recurrent mutation c.3109C>T to be a founder mutation of Southern Chinese.

## Conclusions

*BRCA-*related breast cancer in this Chinese population is associated with family history and adverse pathologic and prognostic features, with *BRCA2* mutations being more prevalent, but *BRCA1* carriers having more aggressive and tn cancers. Compared with Caucasian populations, the prevalences of *BRCA2* mutations and tn cancer are elevated.

# Abstract: *P030* Genetic Cancer Risk Modifiers in Hereditary Breast and/or Ovarian Cancer Families

**Published:** 2009-09

**Authors:** R.D. Brandao, M.J. Blok, J. van Harssel, A. Romano, C. Schrander–Stumpel, J. Geraedts, M. Zeegers, E.B. Goméz García

**Affiliations:** * University Hospital of Maastricht and; † GROW – School for Oncology and Developmental Biology, University Maastricht, Maastricht, Netherlands and; ‡ School of Medicine, University of Birmingham, Birmingham, U.K

## Introduction

Variability observed in penetrance, age of onset, and site of the tumour, both among and within *BRCA* families, suggests that other low-penetrance genetic variants modify the cancer risk.

## Objectives

To study the effect of 6 polymorphisms, recently described as being risk modifiers of sporadic breast cancer (bc) or ovarian cancer (oc) in *BRCA* families.

## Methods

We recorded the cancer history (tumour site, age of diagnosis) of 548 women (293 carriers, 255 non-carriers) from 125 *BRCA* families (72 *BRCA1*, 53 *BRCA2*). The polymorphisms genotyped were: +331G/A and progins, localized in the progesterone receptor gene, *CASP8* D302H, *CASP8–*652 6Nins/del, *FGFR2* (rs2981582), and *TNRC9* (rs3803662). Familial clustering was taken into account in the statistical analyses.

## Results

Two polymorphisms modified the risk of oc in *BRCA1* and *BRCA2* families: +331 G/A heterozygous genotype increased the risk [odds ratio (or): 2.41; 95% confidence interval (ci): 0.98 to 5.96; *p* = 0.056], and *FGFR2* had a protective effect (or: 0.52; 95% ci: 0.28 to 0.96; *p* = 0.037). *FGFR2* was also significantly associated with increased risk of bilateral bc (or: 2.67; 95% ci: 1.45 to 4.92; *p* = 0.002), whereas *CASP8–*652 6Nins/del had a trend toward a protective effect, restricted to *BRCA1* mutation carriers (or: 0.71; 95% ci: 0.48 to 1.05; *p* = 0.084). Furthermore, progins was significantly associated with bc among the *BRCA2* non-carriers (phenocopies—or: 8.17; 95% ci: 2.17 to 30.76; *p* = 0.002).

## Conclusions

We found evidence that certain polymorphisms involved in hormone-mediated cell proliferation and apoptosis modify the bc and oc risks in *BRCA* families. This may be relevant for an individual assessment of the most suitable preventive option among those currently available for each of those patients.

# Abstract: *P032* Genome-Wide Linkage Analysis of High-Risk Breast Cancer Families from the International *BRCA-*X Consortium

**Published:** 2009-09

**Authors:** M. Southey, F. Odefrey, B. Feng, G. Mann, P. Devilee, D. Goldgar

**Affiliations:** * Melbourne University, Melbourne, Victoria, Australia; † International Agency for Research on Cancer, Lyon, France; ‡ University of Utah School of Medicine, Salt Lake City, Utah, U.S.A.; § Westmead Institute of Cancer Research, Sydney, Australia and; || Leiden University Medical Center, Leiden, Netherlands

Although studies have shown that germline mutations in *BRCA1* and *BRCA2* account only for approximately half of all site-specific familial breast cancer (bc), to date there has been little success in using a linkage approach to identify further bc susceptibility loci. Thus, it seems likely that there are additional high-penetrance loci to be discovered. In 2006, we published the results of a linkage analysis in 149 multiple-case bc pedigrees. Although several regions of interest were identified, no definitive evidence of linkage was found, likely because of genetic heterogeneity. To address this, we have now analyzed a further set of 123 families that were part of an international consortium in familial bc including families from North America (*n* = 53), Europe (*n* = 57), and Australia (*n* = 14). All families had at least 3 cases of bc diagnosed at or before the age of 60 and were previously screened for mutations in *BRCA1* and *BRCA2*. The primary analysis used merlin under a dominant mode of inheritance using the same model as in Smith *et al.,* 2006.

## Results

The linkage analyses identified one region with a conditional heterogeneity logarithm of odds (hlod) of 2.2, and three others with hlods > 1.0. In a combined analysis of the new and existing data, suggestive evidence of linkage was identified on chromosome 4 (hlod = 2.4, α = 0.15). When families were stratified by the presence of bilateral bc or of 5 or more cases of bc, the evidence (or the linkage) was increased (hlod = 3.3, α = 0.30 and hlod = 3.6, α = 0.26 respectively). To further refine the hypothesized locus on chromosome 4, we genotyped a further 12 short tandem repeat markers spanning the linked region. Fine mapping of likely-linked families has narrowed the region likely containing a bc susceptibility gene to 14.6 cM covering approximately 18 Mb of dna. We are currently sequencing the most plausible candidate genes within this region.

# Abstract: *P033* Genetic Variation of Human *MDM4* Confers Increased Risk for Breast Cancer in the Ashkenazi Jewish Population^c^

**Published:** 2009-09

**Authors:** V. Joseph, M. Gaudet, V. Devlin, J. Bhatia, K. Kosarin, G. Bond, K. Offit, T. Kirchhoff

**Affiliations:** * Memorial Sloan–Kettering Cancer Center, New York, New York, U.S.A. and; † Ludwig Institute for Cancer Research, Oxford, U.K

The tp53 tumour-suppressor pathway plays a critical role in the progression of a variety of human cancers. Among the known regulators, *MDM4,* a haploinsufficient homologue of *MDM2,* has been shown to bind and inhibit tp53 protein. As part of a candidate tp53 pathway approach, a statistically significant association with breast cancer (bc) risk was observed at the *MDM4* locus in our recent genome-wide association study (gwas) in the Ashkenazi Jewish (aj) population. We observed a large extent of linkage disequilibrium around the *MDM4* locus in aj bc cases from our aj gwas, with multiple single-nucleotide polymorphisms (snps) significantly associated with bc risk. Analysis of these data revealed a susceptible haplotype and a modestly elevated bc risk [*p* = 0.01; odds ratio (or): 1.2; 95% confidence interval (ci): 1.01 to 1.45]. To replicate these findings, we genotyped a tagging snp for this haplotype (rs2369244) in independent collections of 654 aj bc cases and 1085 aj controls. Similar to the findings in the gwas cohorts, the minor allele homozygotes were significantly enriched in cases as compared with controls (*p* = 0.01; or: 1.19; 95% ci: 1.04 to 1.38). Furthermore, a pooled meta-analysis including the gwas data (total of 1133 bc cases and 1351 controls), and controlling for age, suggests that this snp significantly associates with bc risk in aj population. The association was most apparent for minor allele homozygotes (G/G—or: 1.52; 95% ci: 1.18 to 1.95; *p* = 0.00085). Further replication analysis is being planned as part of an international Breast Cancer Association Consortium involving more than 60,000 bc cases and controls of predominantly European ancestry. Besides ongoing functional experiments, the next phase of the study will address possible gene–gene interactions of *MDM4* rs2369244 with other critical players in this pathway, including *MDM2* snp309 and *TP53* R72P, where genetic variation has also been described to be associated with increased risks for other cancer types.

# Abstract: *P034* Rare, Evolutionary Unlikely Missense Substitutions in *ATM* Confer Increased Risk for Breast Cancer

**Published:** 2009-09

**Authors:** S.V. Tavtigian, A. Hartmann, S. Healey, F. Le Calvez–Kelm, F. Lesueur, D. Babikyan, G.B. Byrnes, J. Hall, S. Sangrajrang, J.L. Hopper, M.C. Southey, I.L. Andrulis, E.M. John, P.J. Oefner, G. Chenevix–Trench

**Affiliations:** * International Agency for Research on Cancer, Lyon, France; † Institute of Functional Genomics, University of Regensburg, Regensburg, Germany; ‡ Queensland Institute of Medical Research, Brisbane, Australia; § Institut Curie, Recherche, INSERM U612, Orsay, France; || National Cancer Institute, Research Division, Bangkok, Thailand; # Centre MEGA Epidemiology, University of Melbourne, Carlton, Victoria, Australia; ** Cancer Care Ontario and Mount Sinai Hospital, Toronto, Ontario, Canada and; †† Northern California Cancer Center, Fremont, California, U.S.A

The susceptibility gene for ataxia telangiectasia, *ATM,* is also an intermediaterisk breast cancer susceptibility gene. However, the spectrum and frequency distribution of *ATM* mutations that confer increased risk of breast cancer have been controversial. To assess the contribution of rare variants in this gene to risk of breast cancer, we pooled data from seven published *ATM* case–control mutation screening studies including a total of 1544 breast cancer cases and 1224 controls with data from our own mutation screening of an additional 987 breast cancer cases and 1021 controls. Using an *in silico* missense substitution analysis that provides a ranking of missense substitutions from evolutionarily most likely to least likely, we carried out analyses of protein-truncating variants, splice junction variants, and rare missense variants. We found marginal evidence that the combination of *ATM* protein-truncating and splice junction variants contribute to breast cancer risk. There was stronger evidence that a subset of rare, evolutionarily unlikely missense substitutions confer increased risk. On the basis of subset analyses, we hypothesize that rare missense substitutions falling in and around the fat, kinase, and fatc domains of the protein may be disproportionately responsible for that risk, and that a subset of these may confer higher risk than do protein-truncating variants. We conclude that a comparison between the graded distributions of missense substitutions in cases versus controls can complement analyses of truncating variants to help identify susceptibility genes, and that this approach will help interpret the data emerging from new sequencing technologies.

# Abstract: *P037* A Mouse Model Reveals a Possible Function for *BRCA1* in Human Uterine Leiomyosarcoma

**Published:** 2009-09

**Authors:** D. Xing, G. Scangas, M. Nita, X. Xu, L. He, Y.J.M. Ioffe, P.J. Aspuria, C.Y. Hedvat, M.L. Anderson, E. Oliva, B.Y. Karlan, G. Mohapatra, S. Orsulic

**Affiliations:** * Massachusetts General Hospital, Boston, Massachusetts; † Cedars–Sinai Medical Center, Los Angeles, California; ‡ Memorial Sloan–Kettering Cancer Center, New York, New York; § Baylor College of Medicine, Houston, Texas and; || Harvard Medical School, Boston, Massachusetts, U.S.A

Uterine leiomyosarcoma (ulms) is a rare gynecologic malignancy with a low survival rate. Currently, there is no effective treatment for ulms. Infrequent occurrences of human ulms hamper the understanding of the initiation and progression of the disease, thereby limiting the ability to develop efficient therapies. To elucidate the roles of the *p53* and *BRCA1* tumour suppressor genes in gynecologic malignancies, we generated mice in which *p53* and/or *BRCA1* can be conditionally deleted using anti-Müllerian hormone type ii receptor (Amhr2)–driven Cre recombinase. Mice with a conditional deletion of *p53* developed uterine tumours that resembled human ulms. A concurrent deletion of *p53* and *BRCA1* accelerated the progression of those tumours. Consistent with the hypothesis that *BRCA1* plays a role in ulms, we have shown that the *BRCA1* protein is absent in 29% of human ulms. We have demonstrated that *BRCA1* promoter methylation is the likely mechanism of *BRCA1* downregulation in human ulms. Our findings provide a rationale for investigating therapies that target *BRCA1* deficiency in ulms.

# Abstract: *P039* Morphologic and Immunohistochemical Correlation of Ovarian Epithelial Dysplasia in Prophylactic Oophorectomies from *BRCA1*/*2* Mutation Carriers

**Published:** 2009-09

**Authors:** G. Chêne, J. Dauplat, N. Lagarde, P. Jaffeux, Y.J. Bignon, F. Penault–Llorca

**Affiliations:** * Service d’anatomopathologie and; † Service de Chirurgie, Centre Jean Perrin; ‡ Département d’Information Médicale, CHU Gabriel Montpied and; § Laboratoire d’Oncologie Médicale, Centre Jean Perrin, Clermont–Ferrand, France

## Objectives

Histopathologic examination of material from prophylactic salpingo-oophorectomies performed in patients at genetic risk has revealed frequent abnormalities interpreted as possible pre-cancerous “ovarian dysplasia” lesions. We sought to study the morphologic features and immunohistochemical expression patterns of neoplasia-associated markers in prophylactically removed ovaries.

## Methods

Morphologic features and immunohistochemical expression patterns of Ki-67, p53, and aldh1 (an enzyme significantly associated with early-stage ovarian cancer) were evaluated in 35 prophylactic oophorectomies from *BRCA1*/*BRCA2* carriers and 62 normal ovaries. Representative slides from formalin-fixed, paraffin-embedded tissue blocks were all read blindly by two gynecologic pathologists. Immunohistochemical staining results were correlated with morphologic findings.

## Results

Mean dysplasia score was significantly higher in the genetic risk group than in controls (9.55 vs. 3.62, *p* < 0.0001). Increased aldh1 expression was observed in prophylactically removed ovaries compared with normal ovaries, and expression patterns of Ki67 and p53 were low in both groups.

## Conclusions

The increased dysplasia score and aldh1 expression in ovaries from *BRCA1*/*2* carriers might be consistent with progression towards neoplastic transformation and could justify the use of the term “dysplasia” or intraepithelial ovarian neoplasia. Ovarian dysplasia may be a pre-malignant, noninvasive histopathologic abnormality that could be an important step in early neoplasia, especially in ovaries from *BRCA1*/*2* carriers.

# Abstract: *P050* Screening and Prevention Utilization in Unaffected *BRCA* Carriers

**Published:** 2009-09

**Authors:** M. Beattie, T. Yu, B. Crawford, M. Metrikin, R. Lee

**Affiliations:** * University of California and; † San Francisco General Hospital, San Francisco, California, U.S.A

## Objectives

The purpose of this research is to carefully quantitate the uptake of screening breast magnetic resonance imaging (mri ), chemoprevention, risk-reducing mastectomy (rrm), and risk-reducing salpingo-oophorectomy (rrso) in unaffected *BRCA* carriers. We also aim to compare predictors of each of these options.

## Methods

We thoroughly surveyed all women who received *BRCA* counselling and testing at University of California–San Francisco in the last 15 years. Using the 2008 Cancer Risk Program survey, *BRCA* testers were queried regarding screening and preventive options, risk perception, cancer worry, knowledge, and opinions regarding screening and prevention.

## Results

The survey response rate was more than 80%, with 1137 returns. Approximately 20% of this population tested positive for a known deleterious *BRCA* mutation, and about half of the *BRCA* positives were unaffected with breast or ovarian cancer. Of the 102 unaffected *BRCA* carriers, 66 had *BRCA1* mutations, and 36 had *BRCA2* mutations. Their median age was 38 years (range: 20–64 years). Risk-reducing mastectomy was chosen by 30% of these women, and rrso was chosen by 58%. The median time between *BRCA* testing and undergoing a risk-reducing surgery was 5 months. Of women with breast tissue at risk, 73% received screening breast mri, and 7% chose chemoprevention (3.5% with tamoxifen, 3.5% with raloxifene). Cancer screening knowledge did not differ between women choosing mri, chemoprevention, or surgeries. Breast cancer risk perception was significantly lower in women who chose rrm as compared with other options (*p* < 0.05), but cancer worry did not differ between option choices. As women aged, they tended to choose the following options, from younger to older age: mri, rrm, rrso, chemoprevention (*p* for trend < 0.05).

## Conclusions

The uptake of screening breast mri in unaffected *BRCA* carriers in this cohort was higher by a factor of approximately 10 than was the uptake of chemoprevention. The median time to rrm and rrso, for women who chose those options, was quite short, at approximately 5 months. It is important to present unaffected *BRCA* carriers with a range of options for screening and prevention, understanding that there is a wide variation of uptake for each option.

# Abstract: *P051* Review of *BRCA1* and *BRCA2* Mutation Screening at Guy’s Hospital, London, U.K

**Published:** 2009-09

**Authors:** J. Campbell, H. Storey, S. Lillis, T. Cullup, J. McCauley, D. Moore, C. Jacobs, G. Pichert, S.C. Yau, S. Abbs

**Affiliations:** * DNA Laboratory, GSTS Pathology and; † Clinical Genetics, Guy’s and St. Thomas’ NHS Foundation Trust, London, U.K

In response to the 2004 U.K. National Institute for Health and Clinical Excellence Guidelines for Familial Breast Cancer, our screening strategy for the *BRCA1* and *BRCA2* genes was changed from screening approximately two thirds of each gene using a protein-truncation test and fluorescent heteroduplex analysis to a complete screen of both genes using sequencing and multiplex ligation-dependent probe amplification (mlpa).

Since May 2006, we have screened 564 new referrals and have found *BRCA1* or *BRCA2* pathogenic mutations in 99 individuals (18%). A variant of unknown significance (vus) has been identified in a further 83 individuals (15%). We have also retrospectively completed a full screen on 738 individuals who previously had a negative two-thirds screen. This involved a significant collaboration with the Clinical Genetics Team who reviewed all individuals seen before January 2000 to ensure that they met current testing guidelines. In this group, *BRCA1* or *BRCA2* pathogenic mutations were identified in 45 individuals (6%), and a vus was identified in a further 48 individuals (7%). Of the 144 (65 *BRCA1*, 79 *BRCA2*) pathogenic mutations identified, 127 (51 *BRCA1*, 76 *BRCA2*) were detected by sequencing, and 17 (14 *BRCA1*, 3 *BRCA2*) by mlpa. A total of 40 *BRCA1* mutations and 44 *BRCA2* mutations have been seen in only 1 individual. Of the 11 *BRCA1* and 12 *BRCA2* mutations that have been identified more than once, most have been seen in only 2 unrelated individuals. However *BRCA1* c.4065_4068delTCAA has been seen in 4 unrelated individuals, duplication of *BRCA1* exon 13 in 3 unrelated individuals, *BRCA2* c.755_758delACAG in 6 unrelated individuals, and *BRCA2* c.7988a>T in 5 unrelated individuals.

In total, 126 different vus have been identified, of which 101 have been seen in only 1 individual. These comprise 68 missense variants (23 *BRCA1*, 45 *BRCA2*), 30 synonymous variants (13,17), and 28 intronic variants (8,20).

# Abstract: *P052* Significant Differences Among Physician Specialties in the Management of *BRCA1* and *BRCA2* Mutation Carriers

**Published:** 2009-09

**Authors:** S. Dhar, P. Cooper, S. Hilsenbeck, P.A. Kelly, C. Noll, T. Wang, S. Plon

**Affiliations:** * Baylor College of Medicine and; † University of Texas Health Science Center, Houston, Texas, U.S.A

## Objectives

The National Comprehensive Cancer Network (nccn) has put forth practice guidelines for hereditary breast and ovarian cancer syndrome (hbocs) management. We here present data on how different physician specialties recommend surveillance for patients who have undertaken genetic testing.

## Methods

We conducted an online survey of Texas ama physicians that included random sampling of 5 different specialties: family medicine (fm), obstetrics and gynecology (og), general surgery (gs), internal medicine (im) and hematology–oncology (ho). In 4 hypothetical case scenarios, the physicians were asked to handle hbocs management of unaffected women ages 40–42 years, in the presence of a familial mutation.

## Results

The survey was completed by 23% of the physicians (*n* = 224). For *BRCA-*negative women, 42% of the physicians (with no clearly significant differences among specialties, *p* = 0.08) recommended surveillance as set forth by nccn. Using an aggregate screening intensity score, physicians clearly recommended more screening for mutation-positive women than for mutation-negative women; however, only about 3% followed nccn guidelines. For *BRCA*-positive women, 89% of ho/og recommended breast mri, compared with 63% of im/fm (*p* < 0.0001); 81% of gs/ho, as compared with 35% of im/fm, recommended prophylactic mastectomy (*p* < 0.0001); and 76% of gs/og/ho, as compared with 45% of im/fm, recommended prophylactic oophorectomy (*p* < 0.0001). Overall, physicians who had ordered *BRCA* testing in their practice recommended more intense management of mutation-positive women than did those that had not (*p* = 0.0059 independent of their specialties) and management that was more intense than specified in the nccn guidelines (*p* = 0.0015).

## Conclusions

Although surveillance guidelines and prophylactic surgical options have been established for *BRCA* mutation carriers, many physicians are not cognizant of these guidelines, including the use of new imaging modalities and appropriate prophylactic surgery. Our study identifies a need to target education of physicians by specialty, so that optimal surveillance and management options can be offered to these high-risk women.

# Abstract: *P053* A Rare Case of Anal Melanoma in a *BRCA1* Mutation Carrier

**Published:** 2009-09

**Authors:** L. Dohany, W. Ducaine, D. Zakalik

**Affiliations:** Beaumont Hospital, Royal Oak, Michigan, U.S.A

## Objectives

Mutations in *BRCA1* and *BRCA2* are associated with an increased risk of breast, ovarian, prostate, and pancreatic cancer. There have been reports of a mild association of *BRCA2* mutations with cutaneous and ocular melanoma. We present a rare case of an anal melanoma in a *BRCA1* Ashkenazi Jewish founder mutation carrier.

## Methods

A 62-year-old male of Ashkenazi Jewish descent presented with symptoms of anal pain and hematochezia. Sigmoidoscopy revealed an anal mass, and biopsy demonstrated invasive malignant melanoma of the anal canal. Staging work-up revealed liver metastases. Family history was significant for a sister who died at age 30 from breast cancer, and a 40-year-old daughter diagnosed with ovarian cancer at age 36 who tested positive for a *BRCA1* mutation, 187delAG. The patient presented for genetic counselling and testing. The patient’s peripheral blood was sent for analysis of the three Ashkenazi Jewish founder mutations: *BRCA1* 187delAG, *BRCA1* 5385insC, and *BRCA2* 6174delT.

## Results

Molecular analysis identified a deleterious mutation, 187delAG, in the *BRCA1* gene. This is a frameshift mutation that results in premature truncation of the *BRCA1* protein at amino acid position 39. This confirms the diagnosis of hereditary breast and ovarian cancer syndrome (hbocs).

## Conclusions

This case represents the first report of an anal melanoma in a *BRCA1* carrier and sheds light on an unusual cancer presentation in patients with hbocs. Earlier studies have shown an association between melanoma and *BRCA2* mutations, but there has been little-to-no evidence of this association in *BRCA1* carriers, especially carriers with Ashkenazi Jewish founder mutations. Detection of the *BRCA1* 187delAG mutation in this patient leads to promising therapeutic options using novel systemic agents for this highly aggressive, potentially lethal malignancy.

# Abstract: *P054* A Rare Case of Ocular Melanoma in a *BRCA1* Mutation Carrier

**Published:** 2009-09

**Authors:** F. Khan, L. Dohany, W. Ducaine, D. Zakalik

**Affiliations:** Beaumont Hospital, Royal Oak, Michigan, U.S.A

## Objectives

Hereditary breast and ovarian cancer syndrome (hbocs) is associated with a mildly increased risk of cutaneous and ocular melanoma. Earlier studies have demonstrated ocular melanoma in association with *BRCA2,* but not *BRCA1,* mutations. We present a rare case of an ocular melanoma and primary peritoneal carcinoma in a *BRCA1* mutation carrier.

## Methods

A 62-year-old Caucasian female presented with an abnormal Papanicolaou smear and underwent a laparotomy, which revealed primary peritoneal carcinoma. Her past medical history was significant for decreased night vision, leading to the diagnosis of a left ocular melanoma (spindle-cell, type B) at age 57 years. She underwent a therapeutic enucleation at that time. Family history is remarkable for early-onset breast cancer in her sister and two nieces, all diagnosed in their 40s. The patient presented for genetic counselling and testing following the diagnosis of primary peritoneal carcinoma. The patient’s peripheral blood was sent for full sequence analysis of the *BRCA1* and *BRCA2* genes. Direct dna sequence analysis was performed on the polymerase chain reaction products corresponding to the entire *BRCA1* and *BRCA2* coding region.

## Results

The molecular analysis identified a deleterious mutation, 2530delAG, in the *BRCA1* gene. This is a frameshift mutation that results in premature truncation of the *BRCA1* protein at amino acid position 808, confirming the diagnosis of hbocs.

## Conclusions

The patient described above developed two rare malignancies, ocular melanoma and primary peritoneal carcinoma. Earlier studies have identified a 2%–3% prevalence of *BRCA2* mutations in younger individuals with ocular melanoma. This case represents the first report of an ocular melanoma in a *BRCA1* carrier and highlights the unusual presentation of cancers in individuals with hbocs.

# Abstract: *P055* Detection and Prevention of Hereditary Breast Cancer

**Published:** 2009-09

**Authors:** D. Farengo Clark, S. Mento, G. Grana

**Affiliations:** * Cooper Cancer Institute, Voorhees, New Jersey and; † Arcadia University, Glenside, Pennsylvania, U.S.A

There is a paucity of data looking at how breast cancers are diagnosed in *BRCA* carriers and the stage in which these women are diagnosed. Our hypothesis is that *BRCA1*/*2* carriers will more often present with a palpable mass than be identified through imaging and that they will be diagnosed with advanced-stage disease that requires traditional chemotherapy. We also wanted to examine the relationship between mutation status and pathology. We conducted a retrospective evaluation of female breast cancer patients who carried *BRCA1*/*2* mutations to determine how women find their cancers and how those cancers are treated. Our study included 66 women who were diagnosed with breast cancer and who carried a *BRCA1*/2 mutation. Almost half the women presented with palpable masses, but only 1/2 were diagnosed by mammographic findings. Surprisingly, of the 66 cancers, 50 were diagnosed at stage 0–iib, and only 5 were diagnosed at stage iii or higher. *BRCA1* cancers were more often triple-negative than were *BRCA2* cancers (42% and 21% respectively). Although these cancers were diagnosed at early stages, 92% of *BRCA1* carriers and 68% of *BRCA2* carriers still were treated with traditional chemotherapy. We feel that, if a woman is presented with this data showing that an impending cancer will more often than not require chemotherapy, she may choose more aggressive management and opt for prophylactic mastectomy. Knowing that *BRCA1* cancers are triple-negative may also change recommendations regarding the use of anti-hormonal agents and leave women with fewer prevention options. All of this information should be presented to women to help them make an informed decision about prevention when they receive results.

# Abstract: *P056* Risk Factors for Carcinoma of the Fallopian Tube in Women with a Germline *BRCA* Mutation and in Sporadic Cases

**Published:** 2009-09

**Authors:** D. Vicus, A. Finch, B. Rosen, I. Fan, L. Bradley, I. Cass, P. Sun, R. Royer, B. Karlan, J. McLaughlin, S.A. Narod

**Affiliations:** * Women’s College Hospital; † University Health Network, University of Toronto and; ‡ Samuel Lunenfeld Research Institute, Toronto, Ontario, Canada and; § Cedars Sinai Medical Center, Los Angeles, California, U.S.A

## Objectives

To evaluate risk factors that may relate to the development of fallopian tube cancer in women with and without a *BRCA* mutation.

## Methods

Subjects with fallopian tube cancer were identified from a large international registry of women that carry a *BRCA1* or *BRCA2* mutation (*n* = 56) and from a population-based study of ovarian and fallopian tube cancer in Ontario, Canada (*n* = 67). *BRCA* mutation status was established for all subjects. All subjects completed a questionnaire about medical history and lifestyle factors. Subjects were matched with controls for date of birth within 4 years, for *BRCA* mutation (negative, *BRCA1*, *BRCA2*), country of residence, and past history of breast cancer (yes/no and matched within 5 years of diagnosis). Using conditional logistic regression, the odds ratio (or) and 95% confidence interval (ci) were calculated for parity, body mass index, oral contraceptive use, tubal ligation, and hormone replacement therapy.

## Results

We studied 105 women with fallopian tube cancer (48 with a *BRCA1* mutation, 10 with a *BRCA2* mutation, and 47 with no identified *BRCA* mutation) and 958 matched controls. Increasing parity was associated with a decreased risk of fallopian tube cancer in non-carriers (trend per birth—or: 0.65; 95% ci: 0.45 to 0.93; *p* = 0.02), in *BRCA1* carriers (or: 0.78; 95% ci: 0.60 to 1.02; *p* = 0.07), and in *BRCA2* carriers (or: 0.44; 95% ci: 0.19 to 1.04; *p* = 0.06), but was significant only for non-carriers. Oral contraceptive use was protective for *BRCA1* carriers (trend per year of use—or: 0.90; 95% ci: 0.82 to 0.99; *p* = 0.03), but was not protective for non-carriers (or: 0.99; 95% ci: 0.88 to 1.10; *p* = 0.80) or *BRCA2* carriers (or: 0.95; 95% ci: 0.82 to 1.11; *p* = 0.53). No significant effects were observed for body mass index, hormone replacement therapy, or tubal ligation.

## Conclusions

Parity and oral contraceptive use were associated with a decreased risk of fallopian tube cancer, but these effects were not observed consistently across all groups stratified by mutation. The risk factors for fallopian tube cancer appear to be similar to risk factors for ovarian cancer.

# Abstract: *P057 BRCA* Mutations, Risk and Outcome of Prostate Cancer

**Published:** 2009-09

**Authors:** D. Gallagher, M. Gaudet, P. Pal, T. Kirchhoff, J. Bhatia, S. Fine, H. Scher, P. Scardino, L. Norton, M. Robson, K. Offit

**Affiliations:** Memorial Sloan–Kettering Cancer Center, New York, New York, U.S.A

## Objectives

To determine clinicopathologic characteristics of *BRCA*-associated prostate cancer in men of Ashkenazi Jewish (aj) ancestry.

## Methods

We determined *BRCA* mutation prevalence in 832 aj men with localized prostate cancer and 435 aj controls, and compared age, stage, prostatespecific antigen, Gleason score (gs), overall survival, and other clinical outcome measures among 26 *BRCA* mutation carriers and 806 non-carriers. Kruskal–Wallis tests were used to compare age of diagnosis and gs, and logistic regression models were used to determine associations between carrier status, prostate cancer risk, and gs. Hazard ratios were estimated using Cox proportional hazards models.

## Results

*BRCA2* mutations were associated with tripled risk for prostate cancer [odds ratio (or): 3.18; 95% confidence interval (ci): 1.52 to 6.66; *p* = 0.002]. *BRCA1* mutation conferred no increased risk. *BRCA2* carriers presented at a younger age (median: 62 years vs. 68.2 years; *p* = 0.05). Adjusting for age, *BRCA2* mutation carriers were more likely than non-carriers to have a Gleason score of 7 or higher [hazard ratio (hr): 2.37; 95% ci: 1.14 to 4.94; *p* = 0.021], but *BRCA1* mutation carriers were not (hr: 0.62; 95% ci: 0.12 to 3.10; *p* = 0.56). After 7254 person–years of follow-up, *BRCA1* and *BRCA2* mutation carriers, as compared with non-carriers, had an increased risk of developing biochemical recurrence (hr: 4.32; 95% ci: 1.31 to 13.62; *p* = 0.016 and hr: 2.41; 95% ci: 1.23 to 4.75; *p* = 0.011 respectively) and castrate metastases (hr: 2.15; 95% ci: 0.28 to 16.31; *p* = 0.46 and hr: 3.01; 95% ci: 1.26 to 7.14; *p* = 0.013). There was a greater risk of death from prostate cancer (hr: 5.16; 95% ci: 1.09 to 24.53; *p* = 0.039 and hr: 5.48; 95% ci: 2.03 to 14.79; *p* = 0.001) for *BRCA1* and *BRCA2* carriers respectively.

## Conclusions

*BRCA2* mutation carriers had an increased risk of prostate cancer, a younger age of onset, and a more aggressive phenotype. Prostate cancer cases with a *BRCA1* or *BRCA2* mutation were characterized by an adverse clinical outcome. These results may affect clinical management of this subset of hereditary prostate cancer.

# Abstract: *P058* Single-Staged Breast Cancer Reconstruction Using the Combined Spectrum Expander/Implant

**Published:** 2009-09

**Authors:** L. Lessard, L. Al-Salmi, A. Izadpanah

**Affiliations:** McGill University Health Centre, Montreal, Quebec, Canada

## Introduction

Breast reconstruction is an important procedure that requires attention to detail for optimal outcome. Spectrum implants (Mentor Corporation, Santa Barbara, CA, U.S.A.) have provided us with the tools to optimally adjust for achieving symmetry and patient satisfaction. Single-staged breast reconstruction with saline implants was first introduced by Becker in 1982. Despite advances in our surgical techniques and prosthesis, the reported complication rate remains high at 28%.

## Purpose

We present the senior author’s experience over a period of 7 years for immediate and delayed breast reconstruction as it relates to implant reconstruction. This is the first reported study of breast reconstruction using Spectrum Expander/Implant with the use of the same retropectoral technique and no alloderm. All implants were saline-filled.

## Methods

A retrospective review analysis of patients undergoing Spectrum implants by the senior author from 2001 to 2008 is presented. Specific attention is paid to factors associated with major complications.

## Results

A total of 44 patients underwent 67 Spectrum breast implant reconstructions. The mean age of the study patients was 42.7 years (standard deviation: ±11.6 years; range: 17–67 years). There was no skin necrosis (0%). Complications requiring reoperation and general anesthesia were capsular contracture (*n* = 10; 14.9%), periprosthetic infection (*n* = 3; 4.5%), and delayed sudden deflation (*n* = 2; 3.0%). Only 1 patient was subsequently judged not suitable for reoperation/reconstruction because of severe neutropenia. The subcutaneous valves were removed under local anesthesia during nipple reconstruction without any complication. Photographic technical tips and final results are presented.

## Conclusions

Use of a versatile integrated all-in-one expander/implant (Spectrum) can achieve great results with minimal complication and reoperation rates.

# Abstract: *P059* Early Experiences of the Calgary Breast Health Program High-Risk Breast Cancer Clinic

**Published:** 2009-09

**Authors:** S. Lupichuk, J. Ng, K. Blaikie, S. Leith, T. Power, B. Walley

**Affiliations:** * Tom Baker Cancer Centre and; † Alberta Health Services–Calgary, Breast Health Program, Calgary, Alberta, Canada and; ‡ Queen’s University, Kingston, Ontario, Canada

## Background

In 2007, funding was obtained to formalize and evaluate the Calgary Breast Health Program high-risk breast cancer clinic, which commenced February 2008. The team included a physician, nurse, psychologist, and clerical assistant. The target client population included individuals at high genetic risk (per medical genetics), women diagnosed with lobular carcinoma *in situ* or atypical hyperplasia, and women who received mantle radiotherapy in childhood or teenage years. The initial appointment involved self-completion of a needs assessment questionnaire, a brief meeting with the psychologist, breast (± ovarian) cancer risk assessment, overview of screening and risk-reduction options, and a nurse-led discussion of lifestyle strategies and signs or symptoms of breast (± ovarian) cancer. The pilot period was completed in March 2009, and the clinic continues to operate under the same model.

## Objectives

To describe the demographic profile of clients; the perceived pre-consultation needs of clients; the recommendations made by the clinic; and the referrals generated.

## Methods

All clients seen from February 2008 through May 2009 who agreed to be contacted regarding further research will be sent a consent package. Pertinent information on consenting clients will be abstracted from charts. Descriptive statistics will be applied to the data obtained.

## Results

By May 31, 2009, the clinic had received 116 referrals, and 73 consultations had been completed. Referrals came from medical genetics (*n* = 57), primary care (*n* = 38), surgery (*n* = 20), gynecology (*n* = 1), and psychology (*n* = 1). Of the referrals not seen, most were referred directly to medical genetics (*n* = 22) or are waiting on a pending appointment (*n* = 6). For consenting clients seen by the clinic, aggregate data describing demographic profile, perceived preconsultation needs, recommendations made, and referrals made are presented.

## Conclusions

The introduction of a formal high-risk breast cancer clinic in Calgary has been a feasible undertaking. Assessment and revision of the current clinic model is ongoing.

# Abstract: *P060* Hereditary Breast/Ovarian Cancer High-Risk Clinic: Our First 12 Years

**Published:** 2009-09

**Authors:** C. Kim–Sing, M. McCullum, J. Pike, K. Swenerton

**Affiliations:** BC Cancer Agency, Vancouver, British Columbia, Canada

## Objectives

To describe the clinic’s first 12 years of experience with high-risk screening for women with confirmed *BRCA1* or *BRCA2* gene mutations.

## Methods

To date, 262 women with *BRCA1*/*2* mutations have seen a clinic physician and nurse specialist for consultation. Prophylactic mastectomy with reconstruction is an option discussed with all women, and high-risk breast screening is offered as the alternative. Screening includes clinical breast exam and imaging every 6 months, with an alternating schedule of breast mri (from age 25) and mammography (from age 30). Prophylactic bilateral salpingo-oophorectomy (pbso) is recommended to all mutation carriers. Ovarian cancer screening is not currently recommended. Women are discharged to their family physician’s care after prophylactic surgeries, and those with a new cancer diagnosis or recurrence are referred to an oncologist.

## Results

Of *BRCA1*/2 mutation carriers attending the clinic, 16% have had at least 1 new cancer diagnosis; 46 new cancers have been detected (26 in *BRCA1-*positive, 20 in *BRCA2-*positive), with 4 women having 2 new diagnoses. Cancers diagnosed include 24 invasive breast cancers, 7 ovarian cancers, 4 cases of ductal carcinoma *in situ* (dcis), 2 cases of lobular carcinoma *in situ* (lcis), 2 fallopian tube (ft) cancers, 2 peritoneal cancers (both after pbso), 2 pancreatic cancers, 1 malignant melanoma, 1 gastric cancer, and 1 colorectal cancer. Of the breast cancers, 10 were identified on mri screening. Six cancers were diagnosed by pathology review at the time of prophylactic surgery, including 3 ovarian cancers, 1 ft cancer, 1 breast cancer, and 1 lcis, with an additional dcis diagnosis on contralateral mastectomy.

## Conclusions

Over the past 12 years, the clinic has provided a valuable service to high-risk women in our province. Experienced clinicians offer information, support, referral to expert surgeons, breast imaging, and clinical follow-up. This high-risk clinic will provide ongoing opportunities to evaluate the efficacy of specific cancer risk management strategies.

# Abstract: *P061* Somatic Complaints in Women with Risk-Reducing Salpingo-Oophorectomy to Prevent Hereditary Breast/Ovarian Cancer

**Published:** 2009-09

**Authors:** T.M. Michelsen, A.A. Dahl, C.G. Tropé, A. Dørum

**Affiliations:** * Department of Gynecology, Sørlandet Hospital Arendal, Arendal; † Rikshospitalet, Oslo University Hospital and; ‡ Faculty Division; § Department of Clinical Cancer Research and; || Section of Gynecological Oncology, The Norwegian Radium Hospital, Oslo, Norway

## Background

Risk-reducing salpingo-oophorectomy (rrso) is effective for preventing ovarian cancer in women from hereditary breast/ovarian cancer families, but the procedure may be associated with somatic morbidity and complaints. In this cross-sectional study, we aimed to study self-reported somatic complaints in women who had undergone rrso.

## Methods

Based on surgical records, we identified and invited 503 women who had undergone rrso after genetic counselling at a median of 6 years earlier. Among these, 338 (67%) completed questionnaires on demographic and medical issues, somatic complaints, and mental distress. We randomly allocated 5 age-matched controls per case (*n* = 1690) among participants with intact ovaries from the population-based Norwegian Nord-Trøndelag Health Study.

## Results

The rrso group had significantly more palpitations (*p* = 0.02), constipation (*p* = 0.01), pain and stiffness (*p* = 0.02), musculoskeletal disease (*p* = 0.01), and osteoporosis (*p* = 0.02) than did the control group. In a multivariate analyses of the total sample, membership in the rrso group was significantly associated with osteoporosis [odds ratio (or): 2.4; 95% confidence interval (ci): 1.3 to 4.5], musculoskeletal disease (or: 1.7; 95% ci: 1.0 to 2.7), osteoarthritis (or: 2.4; 95% ci: 1.7 to 3.5), and constipation (or: 1.4; 95% ci: 1.1 to 2.0). Current use of hormonal replacement therapy (hrt) was significantly associated with musculoskeletal disease (or: 1.5; 95% ci: 1.0 to 2.3), osteoporosis (or: 2.0; 95% ci: 1.2 to 3.3), pain and stiffness (or: 1.4; 95% ci: 1.1 to 1.8), fibromyalgia (or: 1.6; 95% ci: 1.1 to 2.5), nausea (or: 1.5; 95% ci: 1.1 to 2.1) and constipation (or: 1.5; 95% ci: 1.2 to 1.9). In multivariate analyses of the rrso group only, no significant association was observed between the use of hrt and somatic complaints, but time since rrso was significantly associated with osteoporosis (or: 1.2; 95% ci: 1.1 to 1.3), nausea (or: 0.9; 95% ci: 0.8 to 1.0), and constipation (or: 0.9; 95% ci: 0.9 to 1.0).

## Conclusions

The rrso group had more palpitations, constipation, and musculoskeletal disease than did the control group. Use of hrt was associated with musculoskeletal pain conditions and gastrointestinal symptoms, which may indicate that women with more symptoms choose hrt. Follow-up examinations after rrso should also focus on somatic complaints.

# Abstract: *P063* Effect of Reminder Telephone Calls on Mammography Compliance in High-Risk Women

**Published:** 2009-09

**Authors:** C. Snyder, H. Lynch

**Affiliations:** Creighton University, Omaha, Nebraska, U.S.A

The effect of a reminder telephone call intervention designed to encourage mammography has not been systematically studied in a high-risk population in which compliance is crucial. We hypothesize that a simple reminder telephone call would significantly increase mammography uptake in high-risk women as compared with a control group. In 447 high-risk women, consent to participate in the study was obtained. Interestingly, 346 (77%) of these women self-reported being compliant in obtaining annual mammography for the preceding 2 years. Verification was established in a subset of these women by obtaining their mammogram reports. Subjects who were noncompliant by self-report (*n* = 32) were randomized to the intervention or the control group. Reminder and follow-up telephone calls were completed on 16 women in the experimental group, and follow-up calls were completed on 15 women in the control group. One woman was excluded from the study after being diagnosed with breast cancer upon obtaining a mammogram. A statistical difference (*p* = 0.0017) was observed between the two groups in support of the hypothesis that mammography compliance in high-risk women can be increased if an intervention such as a simple reminder call is implemented. Future studies should identify barriers in obtaining mammograms in these high-risk individuals.

# Abstract: *P064* Results of a Phase II Open-Label Nonrandomized Trial of Cisplatin Chemotherapy in Patients with *BRCA1-*Positive Metastatic Breast Cancer

**Published:** 2009-09

**Authors:** T. Byrski, R. Dent, D. Zuziak, M. Foszczyñska–Kloda, J. Gronwald, T. Huzarski, C. Cybulski, J. Lubiñski, S.A. Narod

**Affiliations:** * Department of Genetics and Pathology, International Hereditary Cancer Center, Pomeranian Medical University, Szczecin, Poland; † Sunnybrook Odette Cancer Centre, University of Toronto, Toronto, Ontario, Canada; ‡ Regional Oncology Hospital, Bielsko Biala and; § Regional Oncology Hospital, Szczecin, Poland and; || Women’s College Research Institute, University of Toronto, Toronto, Ontario, Canada

## Purpose

To evaluate the efficacy of cisplatin chemotherapy in *BRCA1* mutation carriers with metastatic breast cancer.

## Design

In a phase ii open-label study, 20 patients with metastatic breast cancer who carried a mutation in *BRCA1* were treated with cisplatin 75 mg/m^2^ intravenously every 3 weeks as part of a 21-day cycle for 6 cycles. Restaging studies to assess response were performed after cycles 2 and 6, and every 3 months thereafter.

## Results

Between July 2007 and January 2009, 20 patients were enrolled. At baseline, 65% had received prior adjuvant chemotherapy, and 55%, prior chemotherapy for metastatic breast cancer. Mean age in the group was 48 years (range: 32–70 years); 30% were estrogen (er) or progesterone receptor (pr)–positive; 70% were er/pr/her2–negative; and 0% were her2-positive. Overall response rate was 80%; 9 patients experienced a complete clinical response (45%), and 7 experienced a partial response (35%). The 1-year survival was 93%. Cisplatin-related adverse events, including nausea (50%), anemia (5%), and neutropenia (35%), were mostly mild to moderate in severity. One patient discontinued therapy because of grade 4 neutropenia.

## Conclusions

This phase ii study demonstrates that cisplatin chemotherapy has high activity in women with a *BRCA1* mutation and metastatic breast cancer and is generally well tolerated.

# Abstract: *P065* Outcomes and Uptake of Risk-Reducing Salpingo-Oophorectomy in Women Over the Age of 40 Years at High Familial Risk of Ovarian Cancer

**Published:** 2009-09

**Authors:** R. Manchanda, A. Abdelraheem, M. Johnson, M. Brunell, A. Rosenthal, S. Gessler, C. Brunell, E. Benjamin, D. Oram, I. Jacobs, U. Menon

**Affiliations:** * Gynaecological Oncology, Institute for Women’s Health, University College London; † Barts and the London Hospital; and; ‡ Departments of Pathology and; § Radiology, University College London Hospital, London, U.K

## Objectives

To evaluate outcomes and factors affecting uptake of risk-reducing salpingo-oophorectomy (rrso) in women at high risk for ovarian cancer over the age of 40 years.

## Methods

Prospectively collected data of women attending a Familial Cancer Clinic between 2004 and 2009 were analyzed. The preferred management option recommended to women over the age of 40 years with a 10% or greater estimated lifetime risk of ovarian cancer was rrso with peritoneal washings. Those declining surgery opted for screening under the United Kingdom Familial Ovarian Cancer Screening Study. A strict histopathologic protocol with serial slicing was used for rrso specimens.

## Results

Of 1518 high-risk women from breast/ovarian cancer families seen from 2004 to 2009, 517 were below 40 years of age. Of the 1001 others, 181 were *BRCA1*/*2* carriers, and 820 had unknown mutation status (ums). Of the 1001 women, 263 (of whom 33.5% were carriers) chose rrso, and 738 (of whom 12.6% were carriers) preferred screening.

The median ages (and interquartile ranges) for the rrso and screening groups were 50.9 (45.3, 57) years and 47.5 (43.2, 54.9) years respectively (*p* = 0.001). Women undergoing rrso were more likely to have had breast cancer (*p* < 0.0005), to have a relative below the age of 50 years with ovarian cancer (*p* = 0.012), to have a parity of P2 or greater (*p* = 0.039), to be postmenopausal (*p* < 0.0005), and to carry *BRCA* gene mutations (*p* < 0.0005). The median age of women undergoing rrso was lower in *BRCA* carriers than in ums women (*p* < 0.000001).

The occult cancer rate in the whole cohort was 3.8% [95% confidence interval (ci): 1.84% to 6.88%]. Of the 7 tubal and 3 primary ovarian cancers, 6 were in mutation carriers, for a 6.8% incidence (95% ci: 2.5% to 14.25%), and 4 were in ums women for a 2.3% incidence (95% ci: 0.63% to 5.75%; *p* = 0.09).

## Conclusions

Various factors affect decision-making in high-risk women. *BRCA* carriers and ums women both have a high occult malignancy rate, and the age at detection of lesions is similar in both groups. It is important that women are made aware of these facts, because ums women tend to delay surgery till becoming postmenopausal.

# Abstract: *P070* Increasing Medical Student Awareness of Inherited Susceptibility to Breast Cancer

**Published:** 2009-09

**Authors:** J.S.A. Wronzberg, S.M. Nikkel, E.M. Tomiak

**Affiliations:** * Faculty of Medicine, University of Ottawa; † Department of Genetics, Children’s Hospital of Eastern Ontario and; ‡ Women’s Breast Health Centre, The Ottawa Hospital, Ottawa, Ontario, Canada

In 2007, it was reported that North American medical school curricula are lacking in the area of clinical genetics (Thurston VC *et al. Acad Med* 2007;82:441–5). Implementation of an online self-directed learning module (slm) provides students with an interactive case-based approach that simulates real-world uses of genetic testing and highlights the implications of such tests on patient decision-making.

## Objectives

To design and implement a slm for medical students to improve their knowledge of hereditary cancers using breast cancer as the model.

## Methods

The slm will be designed by a medical student working together with a clinical geneticist and a medical oncologist. The objectives for the module are focused on providing students with knowledge in three areas:

- How to interpret a patient’s personal and family history to assess level of risk
- Understanding the implications of genetic testing as they relate to hereditary cancers
- Increasing awareness of the diversity of psychosocial issues facing women and their families who are found to have a *BRCA* mutation

## Results

Assessment of the efficacy of this slm as a teaching tool will be carried out using a focus group of medical students. The focus group will examine the effectiveness of the slm in increasing student knowledge and in addressing the limitations of the modality so that those limitations can be overcome.

## Conclusions

slms present an excellent opportunity to provide early impact in medical school curricula. By integrating knowledge from several key disciplines (genetics, oncology, and gynecology) with appropriate emphasis on the psychosocial aspects of risk assignment, medical students will have an increased understanding of adult-onset genetic conditions.

# Abstract: *P071* Personal Genetics and Cancer Prevention: Mapping Public Representations and Psychosocial Response to Testing

**Published:** 2009-09

**Authors:** C. Ouellette, R. Hyde–Lay, T. Caulfield

**Affiliations:** Health Law Institute, Law Centre, University of Alberta, Edmonton, Alberta, Canada

It has been suggested that the age of personal genetics is here (Hudson, 2009; Walsh, 2009). Indeed, a service offered by the personal genetics company, 23andMe, was named invention of the year for 2008 (Hamilton, 2008). And in the same year, the investigation of genetic variation was deemed the scientific breakthrough of the year by *Science* magazine. The entire field also gets a tremendous amount of media attention, garnering almost daily coverage in the popular press.

One of the foundational rationales for this emerging field—and the reason for much of the excitement–is that it will allow for a more holistic and preventive approach to the management of chronic diseases, including many forms of cancer (Scheuner *et al.*, 2008). This rationale can be found in both the academic literature and in the marketing strategies of companies offering direct-to-consumer genetic tests. However, current research regarding psychosocial response to genetic testing is mixed, clouding the preventive rationale for the field.

Our study contrasts public representations of risks and benefits with behaviour research. Firstly, we map the psychosocial risks and benefits associated with the emerging field of personal genetics, as communicated through public representations. Secondly, we critically assess the public representation of personal genetics in relation to literature on cancer prevention strategies and its role as a means of health communication. In response to personal genetic testing, how are cancer prevention strategies framed? Do the representations support a preventive approach to personal genetics? How might public representations be improved to promote preventive behaviour in response to testing? Our study hopes to provide insight into the current state of public communications and psychosocial response to genetic testing.

# Abstract: *P080* Secondary Use of Data and Samples from Deceased Participants: What Are the Legal and Ethical Constraints?

**Published:** 2009-09

**Authors:** K. Bédard, E. Lévesque, D. Avard, J. Simard

**Affiliations:** * Université de Montréal and; † Centre of Genomics and Policy, McGill University, Montreal and; ‡ Faculté de médecine, Université Laval, Quebec City, Quebec, Canada

## Context

Biologic samples and data used in research may, in some cases, be of great use for another research project. This is called “secondary use.” The secondary use of data and samples is current practice among health care researchers, even more so since the increasing use of biobanks. However, particular issues arise when the participant to whom the samples and data are linked is deceased. Although ethical and legal framing of secondary use of samples and data of live participants is in place and is consistent across various normative texts, the framework concerning deceased participants is often less developed and sometimes inexistent. Although the value of these samples is often inestimable, using them may sometimes prove to be complex.

## Methods

Our research consists of an analysis of the legal and ethical framework in place in Quebec and Canada concerning the secondary use of data and samples from deceased individuals. We analyzed how the various norms address some specific questions such as the scope of consent for use after death and considerations concerning the wishes of living relatives related to the use of data and samples of a deceased individual, when there is no prior indication of individual choice.

## Results

We will provide a synthesis of the elements that need to be taken into account before considering the secondary use of such material. We will examine, by way of example, the procedure set in place to allow secondary use in the case of the continuation of the inherit breast cancer project, funded by the Canadian Institutes of Health Research Team in Familial risks of Breast Cancer. Finally, we will conclude by presenting lessons learned, which could be helpful for the elaboration of a uniform ethical and legal framework in Canada.

# Abstract: *P081* Breast Cancer at Less Than 36 Years of Age Is a Strong Predicator of *BRCA1*/*2* Status in French-Canadian Cancer Families

**Published:** 2009-09

**Authors:** L. Cavallone, S.L. Arcand, C.M. Maugard, A.M. Mes–Masson, P. Ghadirian, D. Provencher, P.N. Tonin

**Affiliations:** * Department of Human Genetics, McGill University; † The Research Institute of the McGill University Health Centre; ‡ Centre Hospitalier de l’Université de Montréal (chum); § Département de médecine, Université de Montréal; || Centre de Recherche du chum, Institut du Cancer de Montréal; # Epidemiology Research Unit, Research Centre, chum–Hôtel-Dieu; ** Département d’obstétrique gynécologie, Université de Montréal and; ‡‡ Department of Medicine, McGill University, Montreal, Quebec, Canada

The presence of a breast cancer case diagnosed before 36 years of age is a strong indicator for the presence of a *BRCA1*/*2* mutation in French-Canadian (fc) hereditary breast and/or ovarian cancer (hbc/hboc) families. Earlier studies were limited by screening for the mutations that recur in this founder population. We have assessed the contribution of *BRCA1*/*2* to 75 hbc and 51 hboc fc families who have at least 3 cancer cases per family and who have undergone Myriad Genetics mutation screening and multiplex ligation-dependent probe amplification analysis for rearrangements. About 50% of the hbc families were mutation-negative. About 32% were 3-case families, in which 71% were mutation-negative (*p* = 0.014). The distribution of cases relative to an index tested case in the hbc mutation-negative group differed from that in the mutation-positive group (*p* = 0.004), where more first-degree relatives with breast cancer were observed in the mutation-negative families (56%) than in the mutation-positive families (35%, *p* = 0.0009). The frequency of breast cancer diagnosed before the age of 36 years in the hbc mutation-negative families (6%) differed significantly from that in the *BRCA1* (23%, *p* = 0.0002) and *BRCA2* (23%, *p* < 1x10^5^) mutation-positive hbc families. Thus, although 50% of the hbc families harboured a mutation, about 80% of families with at least 1 breast cancer case diagnosed below the age of 36 years were mutation-positive. About 69% of the hboc families were mutation-positive, and as compared with the 19% of mutation-negative families, 54% of those mutation-positive families contained at least 1 breast cancer case diagnosed below the age of 36 years (*p* = 0.017). Of the 18 hboc families with fewer than 3 cases of breast cancer, half of the 10 mutation-positive families contained at least 1 breast cancer case diagnosed below the age of 36 years as compared with no cases diagnosed in mutation-negative hboc families in this sub-group. These observations reaffirm the significance of the presence of very young age of diagnosis of breast cancer with hbc and hboc families and should facilitate decisions aimed at genetic testing for *BRCA* mutations.

# Abstract: *P082* Transitional-Cell Ovarian Carcinoma Is Associated with Germline *BRCA1*/*2* Mutations

**Published:** 2009-09

**Authors:** L.M. Chen, J.T. Rabban, B. Crawford, J. Hwang, J.K. Chan, C.B. Powell

**Affiliations:** UCSF Helen Diller Family Comprehensive Cancer Center, San Francisco, California, U.S.A

## Objectives

The population prevalence of *BRCA1* and *BRCA2* mutations for women with epithelial ovarian carcinoma is estimated to be around 11%–15%, with the predominant histology of tumours being serous. Our study is designed to estimate the strength of the association between transitional-cell ovarian carcinomas and germline mutations in *BRCA1* and *BRCA2*.

## Methods

We searched our pathology department’s database for cases of epithelial ovarian carcinoma with transitional-cell histology. Patients (or their surviving first-degree relatives) were offered genetic counselling and testing for germline mutations in *BRCA1* and *BRCA2*. Pathology slides were reviewed and confirmed by a gynecologic pathologist.

## Results

Between 1996 and 2008, 25 patients with a mean age of 58 years (range: 39–75 years) were identified. Ten patients and one patient’s son underwent genetic testing. The other patients could not be tested because they were either deceased or lost to follow-up. We identified 6 *BRCA1* and 2 *BRCA2* mutation carriers with a mean age of 57 years (range: 51–63 years). A conservative estimate of the prevalence of *BRCA1* and *BRCA2* mutations, including all 25 transitional-cell histology patients as the denominator, identified a frequency of 32% (95% confidence interval: 14%–50%). If we had been able to offer testing to all patients in our transitional-cell cohort, the prevalence of *BRCA1*/*2* mutations may have been even greater. All patients who tested positive had a family history of cancer, including 5 families with breast cancer, 3 with ovarian cancer, and 2 with colon cancer.

## Conclusions

Transitional-cell histology apparently has a higher prevalence of *BRCA1*/*2* mutation carriers than is found in the general population. Further molecular characterization may elucidate whether the transitional-cell and serous histologies represent distinct phenotypes.

# Abstract: *P083* Long-Term Stability of Knowledge Acquired During Genetic Counselling for Breast/Ovarian Cancer Susceptibility

**Published:** 2009-09

**Authors:** M. Dorval, K. Bouchard, C. Côté, S. Camden, J. Simard

**Affiliations:** * Faculty of Pharmacy, Laval University; † URESP, Centre de recherche du cha; ‡ Faculty of Medicine, Laval University and; § Laboratoire de génomique des cancers, Centre de recherche CHUL, Quebec City, Quebec, Canada

## Purpose

Studies have shown that standard genetic counselling increases knowledge of breast and ovarian cancer genetics. However, little is known about the retention of such information long after test result disclosure. This study assesses the stability of knowledge acquired during pre-test genetic counselling up to 3 years following *BRCA1*/*2* test result disclosure.

## Methods

Participants were a consecutive series of 429 French-Canadian women (89 carriers, 120 non-carriers, and 220 with an inconclusive test result) from 187 families, who were tested for *BRCA1*/*2* mutations as part of the inherit brcas multidisciplinary research program. Knowledge about *BRCA1*/*2* testing was assessed by a 19-item true–false measure at the pre-test genetic counselling session and 3 years after result disclosure. Knowledge scores are expressed as percentages of correct answers.

## Results

Three years post-disclosure, mean knowledge scores were moderate (56.7 ± 18.2) and lower than those at pre-test genetic counselling [58.9 ± 18.7; paired *t-*test *p* = 0.0059 (intra-class correlation coefficient: 0.58)]. An average of 70% of participants who answered correctly to a given item at pre-test also answered correctly at 3 years. In multivariate models, higher education (*p* = 0.02) and older age (*p* = 0.04) were associated with a lower retention of information acquired during pre-test genetic counselling. However, women’s retention of information was not affected by test result, cancer status, psychological distress, or elapsed time between pre- and post-measurements.

## Conclusions

Although knowledge acquired during a standard *BRCA1*/*2* genetic counselling session could be improved, information correctly learned was remembered for an extended period of time by most participants.

# Abstract: *P084* A Study of Preventive Practices Among Canadian Women with a *BRCA1* or *BRCA2* Genetic Mutation

**Published:** 2009-09

**Authors:** M. Enmore, C. Kim–Sing, S. Armel, L. Bordeleau, D. Gilchrist, E. Warner, A. Chudley, E. Lemire, P. Ghadirian, S. Narod, K. Metcalfe

**Affiliations:** * Women’s College Research Institute, Toronto, Ontario; † BC Cancer Agency, Vancouver, British Columbia; ‡ Princess Margaret Hospital, Toronto, Ontario; § Juravinski Cancer Centre, Hamilton, Ontario; || Mount Sinai Hospital, Toronto, Ontario; # University of Alberta Hospital, Edmonton, Alberta; ** Sunnybrook Health Sciences Centre, Toronto, Ontario; †† Children’s Hospital, Winnipeg, Manitoba; ‡‡ Royal University Hospital, Saskatoon, Saskatchewan; §§ Hotel-Dieu (CHUM), Montreal, Quebec, Canada

## Background

Women with a *BRCA1* or *BRCA2* mutation may elect not to undergo preventive treatment options despite their availability. In this study, we examined Canadian women’s preventive practices and the reasons that high-risk women elect not to adopt cancer preventive options.

## Methods

Eligible patients were identified from a database of *BRCA1* and *BRCA2* mutation carriers. A study-specific questionnaire was mailed to each eligible woman. The questions focused on uptake of prophylactic mastectomy, prophylactic oophorectomy, chemoprevention, and screening. Reasons for not electing an option were also examined.

## Results

Of the 625 eligible women contacted, 464 (74%) women returned completed questionnaires, 12 (2.6%) of whom underwent no preventive options. The women’s mean age was 51.6 years (range: 25–70 years), and they received their genetic test results a mean of 79.2 months (range: 18–239 months) before completing the questionnaire. Of the 218 women without breast cancer, 147 (67%) opted against prophylactic mastectomy, because of body image and sexuality concerns, and a preference for other preventive options; 49 (22%) opted against prophylactic oophorectomy because of concerns about medical side effects, menopausal symptoms, and effects on sexuality; 195 (89%) opted against preventive tamoxifen because of concerns about side effects and lack of recommendation; and 49 (22%) opted against preventive magnetic resonance imaging because of lack of access, availability, and recommendation.

## Conclusions

Health care providers should ensure that women with a *BRCA1* or *BRCA2* mutation are presented with all preventive options, should be more proactive in addressing the concerns of these women, and should encourage informed decision-making about suitable preventive options.

# Abstract: *P085* Efficiency of BRCAPRO and Myriad Models and the Use of Mutation Probability Thresholds for *BRCA1*/*2* Mutation Detection: A Retrospective Evaluation

**Published:** 2009-09

**Authors:** E.B. Gomez Garcia, J.J.T. van Harssel, Y. Detisch, R.D. Brandao, A.D.C. Paulussen, M.J. Blok, C.E.P. van Roozendaal

**Affiliations:** * Clinical Genetics, University Hospital Maastricht and; † Research Institute Growth and Development (GROW), University of Maastricht, Maastricht, Netherlands

Considerable differences exist between countries in the mutation probability methods and thresholds used to select patients for *BRCA1* and *BRCA2* genetic testing.

## Objectives

To assess the efficiency of the mutation probability methods brcapro and Myriad, as compared with criteria based on cancer history in the family, to select patients eligible for *BRCA1*/*2* mutation detection.

## Methods

We retrospectively calculated the brcapro and Myriad probabilities in 307 probands who had previously been selected for dna analysis according to a list of eligibility criteria based on number of first- and second-degree relatives affected with breast cancer (bc) and/or ovarian cancer (oc), age of diagnosis, presence of bilateral bc, and bc in men. Until the year 2000, dna analysis was performed using the protein truncation test (ptt). The dna from patients in whom no mutation was initially found using the ptt later on underwent complete genetic screening.

## Results

The ptt detected 21 mutations (6.5%); complete genetic screening identified an additional 32 mutations (10.4%) and 11 unclassified variants (uvs—3.6%). Compared with cancer history, a threshold of 10% with brcapro or with Myriad excluded about 40% of the patients from analysis, including 4 patients with a mutation and probabilities under 10% with both programs. All 4 probands had a *BRCA2* mutation. The brcapro and Myriad methods showed similar specificity at 10% threshold, and overall, brcapro was more sensitive than Myriad. Only 2 of the probands with a uv had probabilities above 20% with brcapro and Myriad.

## Conclusions

The mutation probability models brcapro and Myriad are economically more efficient than are cancer history criteria alone. For the detection of *BRCA1* mutations, brcapro, at a 10% threshold, is equally sensitive; however, for *BRCA2* mutations, cancer history is the most sensitive. Most of the patients with a uv have low probabilities with both methods, which may be an indicator of the lack of pathogenicity of those uvs.

# Abstract: *P087* Is It Time for Population Genetic Testing for *BRCA1* and *BRCA2* in the Jewish Population?

**Published:** 2009-09

**Authors:** K. Metcalfe, A. Poll, R. Royer, M. Llacuachaqui, A. Tulman, P. Sun, S. Narod

**Affiliations:** * University of Toronto and; † Women’s College Research Institute, Toronto, Ontario, Canada

## Introduction

There are 2 founder mutations in *BRCA1* and 1 founder mutation in *BRCA2* that are present in up to 2.5% of Ashkenazi Jewish women. To date, it has not been proposed that the entire female Jewish population be eligible for screening. However, given the high frequency of mutations in the Jewish population at large, a full-population approach may be rational. Current guidelines for genetic testing in Ontario, Canada, stipulate the family history of cancer that must be present to confer eligibility for testing. Little is known about the appropriateness of these guidelines in the Jewish population and the acceptance of genetic testing in this group of women.

## Methods

Eligible subjects were women who self-identified as (Ashkenazi or Sephardic) Jewish, who were between the ages of 25 and 80 years, and who resided in Ontario. Study subjects were recruited through a description of the study that was published in a national newspaper on a single occasion in May 2008. Women were asked to complete a study questionnaire and a family history questionnaire, and to provide a blood or saliva sample. Risks of mutation were estimated for each woman using ibis software.

## Results

The overall mutation prevalence in the 2080 women who enrolled in the study was 1.1% (0.5% in *BRCA1* and 0.6% in *BRCA2*). Of the 22 families, 10 (45%) would have met the criteria for genetic testing for *BRCA1* and *BRCA2* under current guidelines from the Ontario Ministry of Health and long-Term Care. There were no differences in risk of mutation for those with and without a *BRCA1* or *BRCA2* mutation (3.9% vs. 1.2%, *p* = 0.23). However, estimates of mutation risk for women with a *BRCA1* mutation were higher than for those without a mutation (7.6% vs. 1.2%; *p* ≤ 10–4). This was not observed for women with a *BRCA2* mutation compared with those with no *BRCA* mutation (0.8% vs. 1.2%; *p* = 0.32).

## Conclusions

The prevalence of *BRCA* mutations in unselected Jewish women from Ontario, Canada, was lower than previously reported. However, many of the women would not have met the criteria for testing based on family history of cancer. Population screening for *BRCA* mutations should be considered for the Jewish population.

# Abstract: *P088* Recruitment of a Population-Based Sample of Black Women for a Study of Inherited Breast Cancer Using a Cancer Registry–Based Approach

**Published:** 2009-09

**Authors:** T. Pal, S. Vadaparampil

**Affiliations:** Moffitt Cancer Center, Tampa, Florida, U.S.A

## Objectives

We investigated *BRCA1* and *BRCA2* (*BRCA*) mutations in a population-based sample of black women diagnosed with breast cancer below the age of 50 years, because of the disproportionate numbers affected with early-onset breast cancer. Furthermore, because black people are typically underrepresented in genetic research studies, we sought to understand the factors associated with recruitment activities and study participation.

## Methods

Following intuitional research board approvals, the State Cancer registry released demographic, clinical, and contact information on all eligible participants. State-mandated recruitment methods included 2 mailings, 3 weeks apart, with a telephone response card for patients who did not wish to be contacted by telephone. If no patient response was received within 3 weeks of the second mailing, a member of the study team contacted the patient by telephone. Participation involved a genetic counselling session over the telephone, and collection of blood or saliva for *BRCA* testing.

## Results

Of the 209 eligible patients identified by the cancer registry, contact was established with 87, of whom 82 were eligible for study participation. The overall rate of interest in study participation was 80% (including 93% for passive follow-up and 68% for active follow-up), of whom 48 have been consented to date. There were no differences in the clinical and demographic characteristics of participants and nonparticipants.

## Conclusions

This is the first study conducted through a State Cancer registry in which the primary goal was to recruit participants for genetic counselling and testing for inherited breast cancer. Use of active follow-up methods was important in study recruitment. In contrast with many earlier studies, our results suggest that black women with early-onset breast cancer are interested in participating in studies of genetics and breast cancer, indicating that participation rates in black women may be enhanced by carefully considering methods to facilitate participation.

# Abstract: *P089* Critical Evaluation of *BRCA1*/*2* Mutation Detection Rates in Southwestern Ontario

**Published:** 2009-09

**Authors:** K. Panabaker, N. Scanlan, T. Paling, P. Ainsworth, J. Jung

**Affiliations:** London Health Sciences Centre, London, Ontario, Canada

Deleterious mutations in the *BRCA1*/*2* genes have not been identified in high-risk families as often as originally expected. In clinical practice, predictions of *BRCA1*/*2* mutation probability are sometimes made using various statistical models. When such models are applied to the same person, a wide range of probabilities is revealed. Although trying to provide best practice to patients, economic challenges also exist in the health care environment. For these reasons, the Cancer Genetics team at the London Health Sciences Centre critically evaluated our experiences regarding cancer risk assessment and our associated *BRCA1*/*2* mutation detection rate. In Ontario in 2000, a provincial committee established 13 eligibility criteria for hereditary breast/ovarian cancer (hboc) screening based on the medical literature at that time, offering gene analysis to individuals with a perceived 10% or greater risk of having a *BRCA1*/*2* gene mutation. The methodology of this study included a review of all pedigrees, of eligibility criteria, and of genetic test results of individuals (*n* = 1270) who participated in index *BRCA1*/*2* testing from 2000 to 2008. A consistent approach to determining which eligibility criteria were used in each case was pertinent to the analysis. The results revealed that one of the criteria (3 or more cases of breast and/or ovarian cancer, any age), representing 48% (608/1270) of all individuals tested, reported a 12.3% (75/608) overall mutation detection rate. However, a subset of this group (3 or more cases of breast cancer, age over 50 years), representing 31% (189/608) of this category, showed only a 2.6% (5/189) positivity rate. Using a conservatively priced hboc screen ($1500), it takes approximately $56,000 to detect 1 mutation-positive individual in this subset. This finding could represent a significant cost savings to the current service testing program in Ontario. Our next goal is to determine if other cancer genetics programs in Ontario experience similar results in their own region, to provide further evidence to influence provincial decision-making.

# Abstract: *P090* We Are On the Manchester Bandwagon—So how Are We Doing?

**Published:** 2009-09

**Authors:** N. Poplawski, J. Armstrong, K. Ryan, G. Suthers

**Affiliations:** * Women’s and Children’s Hospital, North Adelaide; † University of Adelaide, Adelaide and; ‡ SA Pathology WCH Site, North Adelaide, South Australia, Australia

## Background

The American Society of Clinical Oncology recommends offering *BRCA1*/*2* mutation searching to cancer-affected individuals when the probability of identifying a mutation is 10% or higher (*J Clin Oncol* 1996;14:1730–6). In September 2007, our unit began prospectively triaging familial breast and/or ovarian cancer families for *BRCA1*/*2* genetic testing using a combined Manchester score (combined-MS—*J Med Genet* 2004;41:474–80; *J Med Genet* 2005;42:e39). This method was chosen because of its simplicity; the score can be calculated manually without the need for a computer, and the score can be easily recalculated in an outpatient setting after clarification of a family history.

## Objectives

To retrospectively audit our unit’s *BRCA1*/*2* mutation detection rate, based on the combined-MS.

## Patients and Methods

Between early 1995 and April 30, 2009, our service initiated *BRCA1* and *BRCA2* genetic testing in 939 breast and/or ovarian cancer–affected probands. Using in-house clinical criteria, 827 of the 939 had initially been triaged to *BRCA1*/*2* testing, and a retrospective combined-MS was calculated for these probands; 112 had been triaged to testing using a prospectively calculated combined-MS.

## Results

Pathogenic *BRCA1*/*2* mutations were detected in 152 of 939 probands tested (16.1%). When the combined-MS was considered, pathogenic *BRCA1*/*2* mutations were detected in almost one quarter of probands with a combined-MS above 15 (136/566, 24.0%), but in less than 5% of probands with a combined-MS of 15 or lower (16/373, 4.3%). The probability of detecting a pathogenic mutation increased as the combined-MS increased, with the 10% mutation detection threshold reached at a combined-MS of 16 (based on a best-fit trend line, where *R*^2^ = 0.92).

## Conclusions

Our experience suggests that it is appropriate to triage familial breast and/or ovarian cancer families to *BRCA1*/*2* genetic testing when the combined Manchester score is more than 15.

# Abstract: *P091* Non-Acceptance of an Appointment at the Familial Cancer Clinic: A Preliminary Telephone Survey

**Published:** 2009-09

**Authors:** N. Poplawski, K. Ryan, J. Armstrong, M. Raciti, S. Russell, G. Suthers, D. Trott

**Affiliations:** * Women’s and Children’s Hospital and; † SA Pathology WCH Site, North Adelaide and; ‡ University of Adelaide, Adelaide, South Australia, Australia

## Purpose and Methods

A telephone survey was used to determine why some referred clients do not complete and return pre-clinic forms (pcfs—includes family history form, personal medical record consent, individual medical history form, and clinic location preference form), despite being informed that an appointment will be arranged only after the pcfs are returned.

## Results

A group of 47 clients who failed to return pcfs more than 5 months after receiving them were contacted by telephone (7 men, 40 women). Of the 47, 29 (61%) were referred for assessment of familial breast/ovarian cancer. The non-responders gave 57 reasons for not returning pcfs. Most reasons related to difficulties completing the family history form [*n* = 35 (61%)]. Other reasons included appointment not a priority because of ongoing cancer treatment (*n* = 4), concerns about genetic information (*n* = 4), not understanding why the referral was made (*n* = 2), recent death of a close relative (*n* = 2), and concerns about family confidentiality (*n* = 1).

Two thirds [*n* = 30 (63%)] indicated they wanted an appointment “now” and would return their pcfs (2 men, 28 women; 18 familial breast/ovarian). However, only 13 (43%) returned pcfs in the 6-month period after contact. Almost all reported difficulties completing the family history form [*n* = 28 (93%)].

One third [*n* = 17 (36%)] indicated that they would not return pcfs; 11 did not want an appointment, and 6 did not want an appointment, but would consider re-referral at a later date (5 men, 12 women; 11 familial breast/ovarian). Of the 17, 7 (41%) reported difficulties filling out the family history forms.

## Conclusions

Almost 2 in 3 non-responders want an appointment, but have not returned their pcfs, primarily because of difficulties with the family history form. We need to explore ways to assist completion of family history forms while minimizing the impact on genetic counsellor workload and showing respect for passive indications by non-responders that they do not want an appointment.

# Abstract: *P093* Pre-implantation Genetic Diagnosis for *BRCA1*/*2*: The Patients’ Perspective

**Published:** 2009-09

**Authors:** L.R. Rubin, A. Werner–Lin, K. Hurley, R. Stern, A. Phillips, M. Sagi, K. Offit

**Affiliations:** * New School for Social Research; † New York University; ‡ Memorial Sloan–Kettering Cancer Center and; § Hadassah University Hospital, New York, New York, U.S.A

## Objectives

*BRCA1*/*2* carriers face a 50% chance that mutations will be transmitted to their offspring, and concern about passing on *BRCA1*/*2* is a common reason for genetic testing. Pre-implantation genetic diagnosis (pgd) is a promising technology for *BRCA* carriers. Pre-implantation genetic diagnosis is a type of assisted reproductive technology that involves genetic analysis of embryos obtained through *in vitro* fertilization. Couples can transfer only mutation-free embryos to the mother’s uterus to initiate pregnancy. However, use of pgd for adult-onset cancer susceptibility is also controversial. As health care professionals increasingly become involved in discussions of family planning and reproductive technologies in their work with hereditary breast/ovarian cancer, the viewpoints of patients may provide insight for genetic counsellors and physicians attempting to navigate this ethically complex terrain. We report findings of a qualitative investigation of the views of reproductive-age *BRCA1*/2 mutation carriers regarding pgd, focusing on suggestions for integrating pgd into medical practice.

## Methods

Reproductive-age patients (*n* = 22; 21 women, 1 man; 91% European American), recruited from the clinical genetics service of a national comprehensive cancer centre, participated. Participants view a brief presentation regarding pgd for *BRCA1*/*2* mutations, followed by an in-depth interview about their attitudes toward using pgd to prevent transmission of *BRCA1*/*2* mutations, including their suggestions for discussing pgd in the context of cancer-risk counselling. Interviews are recorded and transcribed verbatim, and analyzed using methods of grounded theory.

## Results

Themes regarding information management—including timing, delivery, and depth—were discussed. Although most participants want some information about pgd, suggesting it is “at least glossed over” in the initial counselling session, many emphasized that information should be limited, maintaining the focus on the patient undergoing testing/counselling.

## Conclusions

*BRCA1*/*2* mutation carriers are interested in learning about pgd; physicians and genetic counsellors must be prepared to discuss this technology. Suggestions are provided for tailoring information to the needs and values of patients.

# Abstract: *P094* To Test or Not to Test: The Influence of Ashkenazi Jewish Ethnicity

**Published:** 2009-09

**Authors:** N. Schonberger

**Affiliations:** Sharsheret, Teaneck, New Jersey, U.S.A

Cancer genetic counsellors are well aware of the increased risk for individuals of Ashkenazi Jewish background carrying a *BRCA* mutation and of the fact that breast/ovarian cancer is more likely to have a genetic basis in Jewish than in non-Jewish families. What they may not be as familiar with, is the existence of a community of mainly religious Jewish women who are resistant to genetic counselling/testing because of unique issues surrounding their religious beliefs. Some Jewish women may find that breast/ovarian cancer treatments and/or genetic testing present religious dilemmas above and beyond the emotional dilemmas that all women confront with cancer. When faced with a diagnosis of breast or ovarian cancer, these women have questions and specific psychosocial concerns that may not be understood by the general cancer community, including genetic counsellors and cancer support groups. Some of their concerns surround issues of community, the Mikvah (ritual bath), and cancer as a stigma affecting marriage prospects. These questions need to be addressed by sources knowledgeable in Jewish lore and practices. A national, not-for-profit organization was formed several years ago to address these unique psychosocial, genetic, and practical issues pertinent to Jewish women living with or at high risk of breast cancer. Acquaintance with this organization and an in-depth description of these issues will be the substance of this poster, so that professionals who meet with resistance on the part of their Jewish patients will have a better understanding of how to give them the support they need within the parameters of Jewish religious customs.

# Abstract: *P095* Ethical Aspects of Pre-implantation Genetic Diagnosis for *BRCA1*/*2*

**Published:** 2009-09

**Authors:** S. SenGupta, K. Fordham, P. Serhal, D. Ranieri, T. Mamas, S. Jaroudi, S. Dhanjal, G. Kakourou, A. Doshi, J. Harper, J. Delhanty

**Affiliations:** * University College London and; † Centre for Reproductive and Genetic Health, London, U.K

## Objectives

Individuals with inherited breast and ovarian cancer face difficult ethical choices when making reproductive decisions. In pre-implantation genetic diagnosis (pgd), embryos created by *in vitro* fertilization are genetically tested and unaffected ones are implanted. This study aimed to determine if couples attempting pgd to exclude *BRCA1*/*2* mutations faced ethical and clinical challenges different from those for other cancer predispositions.

## Methods

Couples referred for pgd for cancer predisposition had a preliminary consultation to outline the process. Applications for treatment licenses were made to the Human Fertilization and Embryology Authority (hfea) for each new disorder and for individual patients with *BRCA1*/*2* mutations. Applications were also made for National Health Service funding.

## Results

Between June 1, 1999, and May 31, 2009, 100 referrals were made for pgd for 15 different cancer-predisposing genes. Among 61 couples who opted for pgd, work-up was started for 41 couples who received funding. Overall, 18 cycles of pgd were completed for 12 couples, resulting in the birth of 5 healthy singletons and 4 ongoing pregnancies (pregnancy rate of 50% (9/18) per pgd cycle started). The largest referral group was for breast and ovarian cancer attributable to *BRCA1* (25 couples) and *BRCA2* (6 couples). However, only 35% (11/31) from this group opted for pgd as compared with 87% (13/15) for *NF1,* 71% (12/17) for *RB1,* and 65% (11/17) for *APC* mutations. Patients with *BRCA1*/*2* mutations refused pgd, because the time taken by the sequential process of funding, protocol development, and hfea licensing interfered with their planned prophylactic surgery. Couples with children rarely chose to have pgd.

## Conclusions

The uptake of pgd for *BRCA1*/*2* is clearly much less than for other cancer predispositions. Our study shows that this finding has as much to do with treatment of the female partner as the fact that *BRCA1*/*2* have incomplete penetrance compared with other cancer predispositions.

# Abstract: *P096* Phenotype of *BRCA1*/*2* Large Genomic Rearrangements

**Published:** 2009-09

**Authors:** K.M. Shannon, G. Chan–Smutko, D. Patel, M. Jacobs Gabree, P.D. Ryan

**Affiliations:** Massachusetts General Hospital Center for Cancer Risk Assessment, Boston, Massachusetts, U.S.A

## Objectives

Most large genomic rearrangements (lgrs) in *BRCA1* and *BRCA2* are missed using conventional sequencing technologies. In the United States, lgrs can be found using the BRACAnalysis Large Rearrangement Test (bart) offered by Myriad Genetics. The bart is performed in parallel with dna sequencing on individuals who meet clinical criteria set by Myriad Genetics. The bart can be ordered separately for individuals who do not meet the established criteria. We were interested in determining how many of our patients that carried an lgr in *BRCA1* or *BRCA2* met the criteria.

## Methods

We performed a retrospective chart review of individuals seen at our center for *BRCA1*/*2* testing after the introduction of this new technology. The family histories of individuals with an lgr were assessed and determined to meet or not meet the clinical criteria set by Myriad for inclusion of bart.

## Results

In 5 individuals, an lgr was detected on bart. All lgrs were in the *BRCA1* gene and included del exon 5, del exons 1–2, del exons 19–20, and exon 14–20del26 kb (2 individuals). In 1 of the 5, cancer history clearly fulfilled the Myriad criteria. In 2 of the 5, cancer histories fulfilled the Myriad criteria only because the genetic counsellor obtained cancer histories on the individual’s great aunts, great uncles, and great grandparents. In 2 of the 5, the individual’s personal and family cancer histories did not meet the clinical criteria set by Myriad.

## Conclusions

Although lgrs are expected to account for a small minority of mutations in *BRCA1*/*2,* the phenotypes of these lgrs are not well understood. Families harbouring an lgr do not necessarily meet Myriad’s stringent criteria. Testing using bart should be considered in all families who are offered *BRCA1*/*2* testing.

# Abstract: *P097* Risk Prediction Models of Breast and Ovarian Cancer: What Are the Social and Ethical Issues?

**Published:** 2009-09

**Authors:** M. Tinker, D. Avard, B.M. Knoppers, J. Simard

**Affiliations:** * McGill University and; † Centre of Genomics and Policy, Montreal and; ‡ Université Laval, Quebec City, Quebec, Canada

Risk prediction for breast and ovarian cancer involves a number of factors, including those that are established (such as family history, current age, age at menarche) and those that are genetic (such as a positive *BRCA1* or *2* gene mutation). The integration of breast cancer risk assessment models that take into consideration gene mutations raises a number of ethical, legal, and social concerns with regard to defining risk and risk categories and communicating risk information to the patient, and with regard to the potential value of the information for family members, the privacy and confidentiality of the information, and the potential misuse of the information by third parties. Thus, utilization of risk assessment models requires awareness of the ethical dilemmas. To gain a better understanding of the information available to the public, policymakers, and health professionals concerning risk assessment models for breast and ovarian cancer, we will review policies and position statements issued by international, regional, and national agencies and the relevant academic literature. We will highlight the differences and similarities between various policy statements with the purpose of elaborating the ethical considerations that could guide the development of risk assessment models.

# Abstract: *P098* Is Increased Susceptibility to Breast Cancer an Extracolonic Manifestation of *MYH* Polyposis? Report of a Canadian Kindred

**Published:** 2009-09

**Authors:** E. Tomiak, E. Smith, K. Boycott

**Affiliations:** Children’s Hospital of Eastern Ontario, Ottawa, Ontario, Canada

## Objectives

Bi-allelic mutations of the *MYH* gene, implicated in base excision repair, are known to cause adenomatous colorectal polyps and are associated with very high colorectal cancer risk. The extracolonic manifestations of *MYH-*associated polyposis coli have not been fully characterized. One previous report suggested a substantially increased breast cancer (bc) risk in female *MYH* mutation carriers (Nielsen *et al. J Med Genet* 2005;42:e54).

## Methods

We describe an extended Canadian kindred in which a wide spectrum of tumour types occurred, including myeloma and colorectal, breast, bladder, and uterine cancers.

## Results

Two previously recognized *MYH* mutations, G382X (1145G>A) and E182X (544G>T), were identified in the male proband who presented with colon polyps, colon cancer (age: 46 years), rectal cancer (age: 67 years), and myeloma (age: 47 years). In 3 of 5 female siblings bcs occurred. Ages of onset were 37 years, 68 years, and 57 and 65 years in 1 sister who presented with bilateral bc and in whom *BRCA1*/*2* testing was inconclusive. Of the remaining 2 sisters unaffected by bc, one died at age 47 with colon cancer. The *MYH* test results for the woman with bilateral bc and her sister who developed breast cancer at age 68, are pending and will be presented.

## Conclusions

Our findings will contribute to the scant literature concerning bc risk in *MYH* mutation carriers. Further studies of the frequency of bc in mono-allelic and bi-allelic mutation carriers are warranted.

# Abstract: *P099* Prevalence of *BRCA1* and *BRCA2* Germline Mutations Among Women with Carcinoma of the Fallopian Tube

**Published:** 2009-09

**Authors:** D. Vicus, A. Finch, I. Cass, B. Rosen, J. Murphy, I. Fan, L. Bradley, P. Sun, R. Royer, J. McLaughlin, B. Karlan, S.A. Narod

**Affiliations:** * Women’s College Research Institute and; † Department of Gynecology Oncology, University Health Network, University of Toronto, Toronto, Ontario, Canada; ‡ Cedars Sinai Medical Centre, Los Angeles, California, U.S.A. and; § Samuel Lunenfeld Research Institute, Toronto, Ontario, Canada

## Objectives

The purpose of this study is to determine the prevalence of *BRCA1* and *BRCA2* mutations among women with carcinoma of the fallopian tube.

## Methods

Two series of women diagnosed with carcinoma of the fallopian tube were studied. The first series of subjects was identified from the Ontario Cancer Registry. Those women were diagnosed between 1990 and 1998 or between 2002 and 2004. The second series was identified from Cedars Sinai Medical Centre, Los Angeles, California, U.S.A. They were diagnosed between 1991 and 2007. Each subject was approached to provide a family history and a blood sample for genetic testing for mutations in the *BRCA1* and *BRCA2* genes.

## Results

We recruited 116 patients with fallopian tube cancer (70 from Ontario, 46 from Los Angeles). A deleterious mutation was found in 36 patients (31.0%), 26 in *BRCA1* (22.4%) and 10 in *BRCA2* (8.6%). A family history of ovarian or breast cancer was positive for 26 women; of these, 15 had a mutation (57.5%). In 19 of the patients with a previous history of breast cancer, 15 (79.0%) had a mutation. Of the women who were diagnosed with fallopian tube cancer before the age of 60 years, 40.0% had a mutation, as compared with 19.2% of the women diagnosed after the age of 60 years.

## Conclusions

Approximately 31% of women with fallopian tube cancer have a mutation in *BRCA1* or *BRCA2*. All patients diagnosed with invasive fallopian tube cancer should be considered candidates for genetic testing.

# Abstract: *P100* Evaluation of a Cancer Risk Educational Interventional Tool for Underserved Women

**Published:** 2009-09

**Authors:** R. Lee, M. Metrikin, M. Beattie, G. Joseph, D. Braithwaite, K. Lamvik, S. Jafarieh, B. Crawford, J. Luce

**Affiliations:** * University of California and; † San Francisco General Hospital, San Francisco, California, U.S.A

## Objectives

The purpose of this study is to evaluate the use of an educational tool for cancer risk education in underserved women. The tool is an audiovisual PowerPoint presentation used as an adjunct to genetic counselling.

## Methods

The Cancer Risk Educational Interventional Tool (credit) is a 15-minute story presentation (available in English and Spanish) showing a high-risk family of mixed race. It uses non-scientific images and language specifically designed for a lower-literacy multi-ethnic population. Participant questionnaires evaluating patient satisfaction, risk perception, knowledge of cancer genetics, and anxiety were administered before and after the intervention and at 1 year of follow-up.

## Results

We recruited 52 women of diverse backgrounds (mean age: 47 years; range: 27–69 years; 58% non-white; 59% college not completed; 11% viewing the presentation in Spanish). Of these participants, 15 reported a previous history of cancer, and 5 went on to receive genetic testing. We found an increase in knowledge following credit, specifically in regard to *BRCA* inheritance, *BRCA-*associated cancer risk, and risk-reducing options (all *p* < 0.05). Reports by 98% of the participants indicated that credit helped them to learn more about cancer risk. Overall, patient anxiety decreased post-credit (*p* = 0.02). Among 36 participants who have been interviewed for long-term follow-up, we found that long-term knowledge overall did not change from post-credit levels (*p* < 0.05). There were no changes in anxiety or cancer worry compared with baseline (both *p* < 0.05).

## Conclusions

The credit successfully increased knowledge regarding breast cancer genetics in this patient population without increasing anxiety or cancer worry in the long term. Our long-term goal is to perform a large-scale evaluation of credit among underserved women at risk of hereditary cancer in public hospitals nationwide. Additionally, we are developing versions aimed at African American and Asian American patients.

# Abstract: *P101* Challenges of Applying Genetic Counselling and Testing in a Middle-Income Asian Country

**Published:** 2009-09

**Authors:** S.H. Teo, E. Thirthagiri, P. Kang, D. Lee, S.Y. Yoon, N.A.M. Taib, M.K. Thong, C.H. Yip

**Affiliations:** * Cancer Research Initiatives Foundation, Sime Darby Medical Centre, Subang Jaya, Selangor and; † University Malaya Medical Centre, Kuala Lumpur, Malaysia

## Objectives

The cost of genetic testing and the limited knowledge about *BRCA1, BRCA2,* and other genes in various ethnic groups has limited testing availability in medium- and low-resource countries, including Malaysia. Malaysia is a multi-ethnic Asian country, composed of approximately 54% Malays, 25% Chinese, 7% Indians, and 14% others. In January 2003, the Malaysian Breast Cancer Genetic Study (MyBrCa) was established to

- determine the prevalence of mutations in breast cancer genes;
- develop statistical methods for the identification of those at risk;
- examine the social, religious, and cultural issues of genetic counselling and testing in this Asian setting.

## Methods

Between January 2003 and December 2008, 901 breast cancer patients participated in MyBrCa. Of these, 305 medium- and high-risk individuals have been analysed for mutations in *BRCA1* and *BRCA2* by sequencing and multiplex ligation-dependent probe amplification analysis. The applicability of the Manchester scoring system and boadicea has also been tested. All patients with deleterious mutations in *BRCA1* or *BRCA2* were provided by genetic counselling by a genetic counsellor and clinical geneticist.

## Results

In 43 unrelated individuals, deleterious *BRCA* mutations were found (22 *BRCA1,* 21 *BRCA2*), only 2 of which were recurrent. In addition, 61 variants of uncertain clinical significance were identified (19 *BRCA1,* 42 *BRCA2*). We found that the predictive power of the boadicea risk prediction model and the Manchester scoring system was better for *BRCA1* than *BRCA2*, but both methods underpredicted the number of carriers in our cohort. We report numerous challenges in providing genetic counselling, including having multilingual counselling sessions, inaccurate family histories, and complex family dynamics (18 of 33 counselled probands were from polygamous/polyandrous families; 18 had 6 to 10 and 10 had more than 10 first-degree relatives).

## Conclusions

Taken together, studies in this typical Asian multi-ethnic population underscore the need for collaborative studies among non-Caucasian populations to validate the role of genetic testing and the use of risk-prediction models in ensuring that the other populations in the world may also benefit from the genomics and genetics era.

# Abstract: *P102* A Randomized Controlled Trial of Population Testing for *BRCA1*/*2* Founder Mutations in the Ashkenazi Jewish Population

**Published:** 2009-09

**Authors:** R. Manchanda, U. Menon, K. Loggenberg, S. Gessler, S. Sanderson, I. Udeh, N. Balogun, J. Wardle, I. Tomlinson, A. McGuire, U. Beller, I. Jacobs

**Affiliations:** * Department of Gynaecological Oncology, EGA Institute for Women’s Health, University College London, London, U.K.; † Mount Sinai University, New York, New York, U.S.A.; ‡ Behavioural Sciences Unit, Department of Epidemiology and Public Health, University College London; § London Research Institute, Cancer Research UK and; || Department of Health Economics, London School of Economics, London, U.K. and; # Department of Gynaecology, Shaare Zedek Medical Centre, Jerusalem, Israel

## Objectives

To compare a systematic population-based approach to genetic testing for cancer-predisposing genes with the current family history–based approach.

## Methods

A randomized controlled trial for *BRCA1*/*2* genes (disease model) in the Ashkenazi Jewish (aj) community (population model), with two arms: population screening (study arm) and family history (control arm). Primary outcomes include *BRCA* founder mutations (fms) detected; acceptability; psychological impact; uptake of screening or preventive strategies; and health economics. Inclusion criteria are age over 18 years and aj ethnicity. Exclusion criteria are first-degree relationship to *BRCA* carriers and past *BRCA* testing.

Recruitment depends on self-referral, and a variety of population-based strategies are used to increase awareness. All participants undergo genetic counselling (group or individual) before making a decision regarding testing. Consenting volunteers provide a blood sample and are subsequently followed at 7 days, 3 months, 1 year, 2 years, and 3 years after the test result to ascertain the benefits and disadvantages of testing. Follow-up questionnaires evaluate attitude, knowledge, satisfaction, psychological impact, quality-of-life, uptake of screening or preventive strategies, lifestyle behaviours, and health economics.

## Results

We present data on our experience of initial participants from the pilot phase of 1000 volunteers. Of the 450 people recruited to date, 31% are men, and 69% are women (median age: 59 years; interquartile range: 45 to 66 years). The post-counselling participation rate exceeds 85%. The founder mutation rate is 2.2% (95% confidence interval: 0.55% to 5.04%). Of these participants, 350 have been randomized, and 3 carriers were detected in the systematic screening arm, and 1 was detected in the family history arm.

## Conclusions

Initial experience suggests that it is feasible to undertake a randomized controlled trial for population-based *BRCA* gene testing. Preliminary results show the presence of carriers in individuals without a strong family history of cancer.

# Abstract: *P110* Genetic Counselling: What Is Important to Spouses of *BRCA1-*/*2-*Positive Women?

**Published:** 2009-09

**Authors:** M. Cohen, P. Kredow, S. Friedman

**Affiliations:** * Vanderbilt University School of Medicine, Nashville, Tennessee; † Center for Psychological Services, Northfield, Illinois and; ‡ FORCE, Tampa, Florida, U.S.A

## Introduction

A limited number of studies have examined the interests and preferences of non-affected spouses in the process of genetic testing and counselling for genetic disorders that have sex-limited expression. Our hypothesis was that non-affected spouses of *BRCA1-* or *BRCA2-*positive women would have specific preferences and needs that were unaddressed. This pilot study ascertained those items.

## Subjects and Methods

A questionnaire was distributed to attendees of the Men’s Facilitated Networking Session, force (Facing Our Risk of Cancer Empowered) meeting. All participants, but 1 male, were Caucasian (11/12); ages ranged from 27 years to 75 years (median age: 48 years). The sample was not socioeconomically representative of the general population: 92% were in professional occupations or retired; 100% had a bachelor’s degree; 42% had advanced graduate degrees.

## Results

In the study group, 100% prioritized genetic counselling before testing and did not differentiate between determining a son’s or daughter’s risk; 92% desired discussion of insurance discrimination issues; 83% preferred receiving results with family members present; 75% felt that determining the cancer risk for self and offspring was very important, wanted a summary letter to share with relatives, and wanted information about local and national support groups, available research protocols, and referrals to other specialists; 50% both desired an additional, separate counselling session for the spouse and offspring without the proband present, and felt that the sex of the health care provider was not important; 25% desired an annual visit with a genetic counsellor.

## Conclusions

Non-affected spouses have unmet preferences and needs. Important needs were insurance discrimination issues, receiving results in person, risks for self and offspring, and a summary letter and referrals to support groups and research programs. The sex of the health care provider and of a child’s future cancer risk were least important. Additional counselling sessions without the spouse present are desired. Genetic counsellors should be prepared to address these preference and needs. Additional research is needed to develop tools for this purpose.

# Abstract: *P111* The Lived Experiences of Women Possessing a Genetic Predisposition to Developing Breast Cancer: A Grounded Theory Study

**Published:** 2009-09

**Authors:** J. DiMillo, A. Samson, S. Lowry, L. Corsini, A. Bodurtha, S. Verma, E. Tomiak

**Affiliations:** * Faculty of Education, University of Ottawa; † Women’s Breast Health Centre, The Ottawa Hospital; ‡ Department of Genetics, Children’s Hospital of Eastern Ontario and; § The Ottawa Regional Cancer Centre, The Ottawa Hospital, Ottawa, Ontario, Canada

## Objectives

Genetic testing to determine whether a woman carries a *BRCA1* or *BRCA2* mutation, which may entail an up to 80% lifetime risk of developing breast cancer, is relatively recent. There is an apparent void in the literature investigating the lived experiences of these women, and qualitative studies of this nature are virtually nonexistent. The purpose of this ongoing study is to better comprehend the lived experiences of young women who have been identified as carrying a *BRCA1*/*2* mutation. In our study, concepts such as the nature of these women’s received support, the impact of the experience, and the presence of stigmatization, are being examined.

## Methods

Six women between the ages of 20 and 40 years who were identified as *BRCA1*/*2* mutation carriers will be invited to participate in a semi-structured interview. The interview will be digitally recorded, transcribed, and analyzed through the use of constant comparison analysis (Glaser and Strauss, 1967).

## Results

This preliminary report is based on the first completed interview. Three major dimensions emerged from 16 raw data themes. More specifically, the themes of Confirmation of Fears, Concern, and Coping were revealed. Generally, this woman was found to experience various negative emotions. The discovery of a positive test result was perceived to be a confirmation of previous fears, which then led to fears for her daughters and about judgments from others. Finally, the woman was able to engage in coping behaviours such as altering her way of life and seeking social support. Results for the remaining 5 planned participants will be presented.

## Conclusions

The results of this qualitative study may lead to recommendations regarding the additional support and psychological assistance that should be considered in young women undergoing genetic testing for inherited predisposition to breast cancer.

# Abstract: *P112* Impact of a Hospital-Based Focused, Counsellor-Led Support Group Among *BRCA* Carriers

**Published:** 2009-09

**Authors:** W. Ducaine, L. Dohany, D. Zakalik

**Affiliations:** Beaumont Hospital, Royal Oak, Michigan, U.S.A

## Objectives

Individuals carrying a mutation in the breast and ovarian cancer susceptibility genes *BRCA1* and *BRCA2* often face significant psychological and emotional challenges. To address the unique medical and psychological needs of *BRCA* carriers, we implemented a hospital-based, focused support group for *BRCA* mutation carriers led by genetic counsellors. This study evaluates the impact of such a support group on the knowledge, emotional well-being, and family dynamics of *BRCA* mutation carriers.

## Methods

We invited 115 individuals carrying a *BRCA* mutation to participate in a voluntary electronic questionnaire about their experience in the support group. Participants were asked to rate the impact of the support group on medical knowledge, emotional needs, family dynamics, and overall benefit.

## Results

The online questionnaire was completed by 42 individuals, 62% of whom were *BRCA1-*positive, 30% were *BRCA2-*positive, and 8% were *BRCA-*negative family members. Most of the responders were between the ages of 40 and 55 years. Of the respondents, 85% felt that attending the support group significantly improved their medical knowledge; 70% felt that the group provided significant emotional support; and 96% felt that they were better able to broach the subject of their *BRCA* status with family members. Slightly more than half of respondents (56%) experienced significantly lowered anxiety levels regarding their *BRCA* status, and a preponderance (78%) reported diminished feelings of isolation since attending the support group.

## Conclusions

Involvement in a focused, interactive *BRCA-*positive support group resulted in significant improvement in medical knowledge, family dynamics, and anxiety in the participants. Genetic counsellors with a specialty in cancer genetics proved to be effective in leading the sessions and achieving its goals. This study demonstrates that ongoing involvement in similar support groups provides *BRCA* carriers with important benefits in dealing with the many challenges faced by this unique group of individuals.

# Abstract: *P113* Decision-Making About Inherited Breast/Ovarian Cancer Risk: Exploring Dimensions of Genetic Responsibility^f^

**Published:** 2009-09

**Authors:** H. Etchegary, F. Miller, S. deLaat, B. Wilson, J. Carroll, M. Cappelli

**Affiliations:** * Eastern Health, Memorial University, St. Johns’s, Newfoundland and Labrador and; † University of Toronto, Toronto; ‡ McMaster University, Hamilton; § University of Ottawa, Ottawa; || Mount Sinai Hospital, Toronto and; # Children’s Hospital of Eastern Ontario, Ottawa, Ontario, Canada

## Background and Objectives

Because genetic information has implications for family members, some choices about genetic risk may be influenced by perceptions of responsibility to relatives. Drawing upon 25 semi-structured interviews with test recipients in Canada, this study explored decisions about inherited breast/ovarian cancer.

## Methods

Semi-structured interviews.

## Results

Qualitative data analysis revealed the pervasive significance of genetic responsibility in test decisions. We highlight three dimensions of genetic responsibility:

- To know about the self for self
- To know about the self for others
- To know about the self to oblige others to know

## Conclusions

These dimensions of genetic responsibility have implications for test decisions, family relationships, and other family members’ desire to know (or not know) and to act (or not act) with respect to their own genetic risk. In particular, genetic responsibility may play out as a framing of a relative’s moral obligation to know their risk that could obviate any interest they might have in not knowing. We conclude that perceptions of responsibility to, and of, other family members be thoroughly explored in genetic counselling sessions.

# Abstract: *P114* Women’s Constructions of the “Right Time” to Consider Decisions About Prophylactic Surgery

**Published:** 2009-09

**Authors:** A.F. Howard, J.L. Bottorff, L.G. Balneaves

**Affiliations:** * University of British Columbia, Vancouver and; † University of British Columbia, Kelowna, British Columbia, Canada

Women who are notified that they carry a *BRCA1*/*2* mutation are presented with surgical options to reduce their risk of breast and ovarian cancer, including risk-reducing mastectomy (rrm) and risk-reducing oophorectomy (rro). There is growing evidence that a subgroup of women do not make decisions about rrm and rro immediately following genetic testing; rather, they consider these decisions years later. Women’s perspectives on the timing of these decisions are not well understood. Accordingly, the purpose of this study was to describe how women construct the “right time” to consider decisions about rrm and rro. In-depth interviews were conducted with 21 women who were *BRCA1*/*2* carriers and were analyzed using qualitative, constant comparative methods. The study findings revealed how important it was for the women to make decisions about rrm and rro one at a time. Moreover, the women constructed the “right time” to consider decisions about risk-reducing surgery to be when

- decisions fit into their lives,
- they had enough time to think about decisions,
- they were ready emotionally to deal with decisions,
- all of the issues and conflicts were sorted out,
- there were better options available, and
- the health care system was ready for them.

These findings offer novel insights relevant to health care professionals who provide decision support to women considering rrm and rro decisions.

# Abstract: *P115* Using Participatory Action Research Techniques, Healthy *BRCA* Mutation Carriers Design a Model of Care

**Published:** 2009-09

**Authors:** P. Mor

**Affiliations:** Shaare Zedek Medical Center, Jerusalem, Israel

## Objectives

To fill a gap in health care and health promotion for healthy women testing positive for *BRCA* gene mutations.

## Methods

Using participatory action research techniques involving a series of focus group interviews and intensive discussions, we worked closely with 23 women recruited from a genetics clinic. The goal was to define unmet care needs for healthy women with *BRCA* gene mutations.

## Results

Through their narratives, carriers described dilemmas that affect and sometimes radically change life perspectives. They identified ongoing stress as a result of the constant threat of illness, knowledge that the mutation can be passed on to children, the need for heightened surveillance, and the difficulty of making decisions regarding prophylactic surgeries and other risk-reducing measures. Inconsistent and inaccurate information from health care providers, barriers to reimbursement for approved screening studies and prophylactic procedures, and a lack of psychosocial support were named as major health care issues. Participants suggested a “one-stop” centre to provide expert care and smooth bureaucratic barriers to screening and preventive procedures. Their ideas were presented to focus groups of health care professionals who manage care for healthy carriers, with the result that a multispecialty outpatient clinic for healthy carriers was opened. The clinic provides improved access to imaging surveillance; expert medical consultation; counselling by a nursing coordinator, psychologist, and sexologist; and medical information. Study findings strengthened interdisciplinary connections among participating nurses, genetic counsellors, and physicians, and supported a holistic health promotion model.

## Conclusions

The primary study outcome was inception of a one-stop multidisciplinary clinic incorporated into an existing health care setting. The clinic provides medical and psychosocial services, including improved access to imaging surveillance, and expert medical consultation and counselling.

# Abstract: *P116* Testing Young People for *BRCA1*/*2*: Challenges from Health Professionals’ Perspective

**Published:** 2009-09

**Authors:** A. Navarro de Souza, N. Wong

**Affiliations:** * McGill University and; † Jewish General Hospital, Montreal, Quebec, Canada

## Objectives

To examine the challenges facing health care providers in deciding to test adult children (individuals 17 years of age and older) for a mutation in *BRCA1*/*2.*

## Methods

A focus group was conducted consisting of 1 medical geneticist and 3 genetic counsellors, who all specialize in cancer genetics and who all have encountered a case in which a young individual requested testing for a mutation in *BRCA1*/*2* known to run in the family. The session was audiotaped. Themes were extracted and examined.

## Results

Recognizing the different arguments in favour of and against testing young people based on theoretical benefits and harms, the group discussed their clinical experience and their case-based decisions. The adult children were mainly female and came with only 1 parent who was not necessarily the carrier. The prominent theme encountered was to elicit the extent to which the young person’s demand for the test was “voluntary,” that is, being “influenced” but not “coercively” by the parents. This assessment became more difficult by the value of autonomy based on a rational individual, traditional bias on psychological assessment, and the limitations of professional training for the changing demands in this select population. Sex differences in the parent’s perspectives on the daughter’s desire to seek testing and contradictory behaviour in relation to being tested or to receiving the result were also relevant themes for the group. An illustrative case in which the resource of a second opinion was used is also presented.

## Conclusions

In the face of the “new generation” seeking predictive testing for *BRCA1*/*2,* this study illustrates the need for more research and training for the professionals who are more often involved with a complex family dynamic rather than an individual choice. Case-by-case decision based on second opinion or psychological assessment in more difficult family interactions can be adequate resources in some cases.

# Abstract: *P117* Breast Cancer Risk Decision: The Experience of Unaffected Carrier Women of Different Cultural Backgrounds

**Published:** 2009-09

**Authors:** A. Navarro de Souza, N. Wong, S. Zaor, D. Groleau

**Affiliations:** * McGill University and; † Jewish General Hospital, Montreal, Quebec, Canada

## Objectives

This study investigated the experience of unaffected women carrying a mutation in *BRCA1* and *BRCA2* and their different choices in strategies to manage breast cancer risk. We are interested in identifying the meaning that women attribute to their test result and to the various risk management strategies, and whether the meaning and experience vary according to culture. To the best of our knowledge, there are no qualitative studies on psychocultural determinants for *BRCA-*mutation carrier women on the uptake of effective cancer prevention options. This aspect is important to investigate because of the observed international and national differences in uptake for the various risk-reducing strategies.

## Methods

A cross-sectional qualitative study is being done. A purposive sample consisting of eligible women is being recruited from a large university-affiliated hospital in Montreal, Quebec. To be eligible for this study, women must be of French-Canadian or Ashkenazi Jewish descent, unaffected at the time of genetic testing, with knowledge of their *BRCA1*/*2* mutation status for at least 3 years, and a decision in favour of, or against, bilateral prophylactic mastectomy made before a diagnosis of breast cancer, if applicable. A theoretically driven, semi-structured qualitative interview protocol designed to elicit illness narratives in health research—the McGill Illness Narrative Interview—is being used. Audiotapes will be transcribed verbatim. We chose the narrative approach to perform a data analysis that allows us to capture individual and collective meanings and the complexities of the experience, which will be contextualized in the cultural backgrounds of the patients.

## Results

Currently, the interviews and analysis are in process, and the results are expected to provide new knowledge on the psychocultural determinants of women’s experience of undergoing *BRCA* genetic testing and women’s decisions on risk-reduction strategies.

# Abstract: *P118* Factors Associated with Choosing Risk-Reducing Mastectomy in *BRCA* Mutation Carriers

**Published:** 2009-09

**Authors:** K. Singh, X. Li, O. Gordon

**Affiliations:** Cedars Sinai Medical Center, Los Angeles, California, U.S.A

## Objectives

Lifetime risk of breast cancer among *BRCA* mutation carriers is markedly reduced with prophylactic mastectomy (pm), yet only a subset elect to undergo the procedure. This study investigates factors that might influence patients’ decisions regarding risk-reducing surgery.

## Methods

We conducted a prospective observational study of *BRCA* mutation carriers with no personal history of cancer presenting to Cedars Sinai Medical Center between 1999 and 2009. Charts were analyzed for factors that might have influenced choices regarding prophylactic surgery, including age, family history, history of breast biopsy, and history of previous cosmetic surgery.

## Results

Of 59 *BRCA* carriers, 22 (37.3%) elected pm. Average time from results disclosure to pm was 1.4 years. In the pm group, as compared with the no-pm group, history of mother dying of breast cancer was 63.6% versus 5.4%, *p* < 0.001, and history of a mother dying of ovarian cancer was 13.6% versus 43.2%, *p* = 0.02. Of those who had pm, 96% as compared with 40.5% in the no-pm group had bilateral salpingo-oophorectomy (bso), *p* < 0.001. Interestingly, 14 of 15 patients (93.3%) who had bso in the no-pm group had a history of mother dying of ovarian cancer. No significant differences in mean age at the time of genetic testing, number of affected first-degree relatives, history of breast biopsy, or history of cosmetic surgery were detected between the groups.

## Conclusions

*BRCA* carriers with family history of a mother dying of breast cancer were most likely to choose pm. Among those who did not undergo pm, uptake of bso was highest among those whose mother died of ovarian cancer. The impact of having a mother die of cancer may be of under-recognized import in patient attitudes regarding prophylactic procedures. Additional studies are needed to support these findings and to assess whether directly addressing this life experience during genetic counselling influences patient behaviour regarding screening versus prophylactic surgery.

# Abstract: *P119* High Satisfaction Rates in Women after Deep Inferior Epigastric Artery Perforator Flap Breast Reconstruction

**Published:** 2009-09

**Authors:** J.P. Gopie, T.H.C. Damen, R. Timman, E.H. Kunst, P.J.C. Bresser, C. Seynaeve, M.B. Menke–Pluijmers, M.A.M. Mureau, S.O.P. Hofer, A. Tibben

**Affiliations:** * Leiden University Medical Center, Leiden and; † Erasmus University Medical Center, Rotterdam, Netherlands and; ‡ University of Toronto, Toronto, Ontario, Canada

## Objectives

Breast reconstruction (br) is aimed at improving quality of life (qol) after mastectomy. Patient satisfaction is an important indicator in evaluating the success of br. This study explored patient satisfaction and its determinants in women undergoing deep inferior epigastric artery perforator (diep) flap br and the impact of the procedure on body image, sexuality, and qol.

## Methods

Patient satisfaction and qol was studied in 72 women who underwent diep flap br, using a study-specific questionnaire and the Short Form 36.

## Results

Patient satisfaction was very high. Approximately 90% of patients reported that they had been sufficiently informed about the procedure and its consequences, that their preoperative expectations had been met, that the reconstructed breast felt like their own, that they would choose the same procedure again, and that they would recommend this procedure to a friend. Patient satisfaction was positively and significantly related to the reconstructed breast or breasts feeling like their own. Women with secondary reconstructions were more positive about changes in sexuality and femininity than were women with primary brs. There were no clinically relevant differences in qol between our study population and a random sample of dutch females.

## Conclusions

Women with diep flap brs reported high satisfaction rates. However, to compare these satisfaction rates with other forms of br, a prospective study in comparable groups is currently ongoing.

# Abstract: *P120* Opening the Psychological Black Box in Genetic Counselling: Recollections and Interpretations of Counselees

**Published:** 2009-09

**Authors:** J. Vos, J. Oosterwijk, E. Gomez–Garcia, F. Menko, C.J. van Asperen, A.M. Jansen, A. Stiggelbout, A. Tibben

**Affiliations:** * Leiden University Medical Center, Leiden; † University Hospital Groningen, Groningen; ‡ Maastricht University Medical Center, Maastricht; § Free University Medical Center, Amsterdam and; || Erasmus Medical Centre, Rotterdam, Netherlands

## Objectives

It is often assumed that the communication of dna test results has direct psychological and medical consequences for the counselee. However, subjective factors may mediate between dna test result and impact. The present study assessed mediation effects of the counselee recollections and interpretations regarding cancer risks and the likelihood that the cancer in their family is heritable.

## Methods

In this retrospective study, women tested for *BRCA1* or *BRCA2* completed questionnaires 5 years after disclosure. Participants had received an unclassified-variant test result (*n* = 76), uninformative-negative dna test results (*n* = 76), or pathogenic mutations (*n* = 51) in *BRCA1*/*2*.

## Results

Of the counselees, 10%–42% correctly recalled and interpreted what the counsellors actually communicated about cancer risks and heredity likelihood. Cancer risks were recalled and interpreted as lower, heredity likelihood as higher. Moderate correlations between recalling and interpreting suggested distinctive processes. Cancer risks were recalled more correctly when measured in percentages instead of in categories. Unclassified dna variants were recalled and interpreted as giving very high cancer risks and heredity likelihood, uninformative results as very low. Psychological and medical outcomes were predicted by cancer risks and heredity likelihood as actually communicated by counsellors. However, these predictions were lowered or became insignificant when the recollections and interpretations of the counselees were included in analyses and path analyses, suggesting mediation. Overestimations predicted life changes, breast/ovarian removal in all counselees, and low quality of life in unclassified variants.

## Conclusions

The impact of test results is strongly mediated by the recollections and interpretations of communicated risks by counselees. Subjective processes should be addressed during counselling, especially if unclassified-variants are disclosed.
